# Decoding m6A: a new frontier in maternal-foetal immunology

**DOI:** 10.3389/fimmu.2026.1770723

**Published:** 2026-03-17

**Authors:** Ruimin Yuan, Junzhe Hao, Mingyu Huang, Yumeng Lin, Haoran Chen, Chuchu Wang, Lan Yuan, Zhongyu Han

**Affiliations:** 1 Chengdu University of Traditional Chinese Medicine, Chengdu, China; 2 School of Acupuncture and Tuina, Chengdu University of Traditional Chinese Medicine, Chengdu, China; 3 School of Pharmacy, China Pharmaceutical University, Nanjing, China; 4 Nanjing Tongren Hospital, School of Medicine, Southeast University, Nanjing, China; 5 Chengdu Xinhua Hospital Affiliated to North Sichuan Medical College, Chengdu, China; 6 The First Affiliated Hospital, Tianjin University of Traditional Chinese Medicine, Tianjin, China; 7 School of Medicine, Southeast University, Nanjing, China

**Keywords:** diagnostic and therapeutic translation, embryonic development, m6A detection technologies, m6A modification, maternal–foetal immune microenvironment, pregnancy

## Abstract

m6A is the predominant internal RNA modification in eukaryotic cells and is distinguished by its abundance and evolutionary conservation. This epigenetic mechanism is dynamically controlled by a coordinated system of writer, eraser, and reader proteins. This sophisticated posttranscriptional regulatory mechanism precisely controls gene expression by influencing RNA metabolism, including its stability, translation, and splicing. Recent advances have revealed the functions of m6A in female reproductive cancers, early embryonic development, and stem cell differentiation. However, its functional roles and molecular mechanisms throughout pregnancy and in related disorders remain incompletely understood, which, to some extent, limits its clinical translation. This review systematically outlines the core regulators of m6A, advanced detection technologies, and its regulatory network across the continuum of pregnancy. Given the immunological parallels between the maternal–foetal interface and the tumour microenvironment, we discuss the possible function of m6A modifications in regulating the maternal–foetal immune microenvironment. The aims of this review were to elucidate the m6A regulatory network across gestation and evaluate its potential as a source of diagnostic biomarkers and therapeutic targets for pregnancy-related pathologies.

## 1 Introduction

The increasing global incidence of pregnancy-related disorders, including gestational diabetes mellitus (GDM), represents a significant concern because of the substantial risks they pose to both maternal and newborn health (1). Despite the continuous progress in related research, the exact pathogenesis of these diseases has not been fully elucidated because of the interactions among genetic, environmental and lifestyle factors, making this problem the focus and challenge of current research.

Against this background, traditional genetics-based research has proven insufficient to fully elucidate the complex pathogeny of these conditions. In recent years, epigenetic regulation, particularly N6-methyladenosine (m6A) modification, has emerged as a novel and promising avenue for deciphering the mechanisms underlying pregnancy-related disorders, owing to its ability to dynamically respond to environmental cues and precisely regulate gene expression. m6A is the main post-transcriptional modification form in eukaryotic mRNA (2). This modification involves the addition of methyl covalent to the N6 position of adenosine to form N6-methyl adenosine. Its dynamic reversibility is controlled by a set of special writer (methyltransferase), eraser (demethylase) and reader (reading protein) to ensure the accurate addition, erasure and decoding of methyl markers, thus guiding downstream functions. Although the core regulatory mechanism has been identified, the specific function of m6A in different physiological and pathological states is still being revealed (3). m6A modification is an important regulatory factor for mRNA metabolism, coordinating multiple stages from transcription and clipping to nuclear output, translation and degradation (4). In addition, m6A modification is widely involved in key biological processes such as spermatogenesis (5), early embryonic development (6), stem cell renewal (7), and immune responses (8).

In view of its multifunctional role, m6A is involved in the precise regulation of female pregnancy. Successful pregnancy depends on a series of closely coordinated events, including fertilisation and embryo implantation, early embryonic development and placental formation, pregnancy maintenance and foetal growth, and homeostasis of the maternal and foetal interface. In these processes, m6A modification plays a key regulatory role (9). In addition, m6A modification is closely related to a variety of pregnancy and reproductive endocrine diseases, including abortion, preeclampsia, GDM, foetal growth restriction, polycystic ovary syndrome (PCOS) and premature ovarian failure (POI) (10–12).

Although the effects of m6A modification during pregnancy have attracted widespread attention, research in this area remains largely fragmented. Specifically, while existing studies have reported altered m6A levels in various pregnancy-related disorders, the underlying dynamic regulatory mechanisms are still insufficiently elucidated. Furthermore, the research perspective has often been confined to individual diseases or specific cell types, failing to situate m6A within the broader physiological and pathological regulatory network of pregnancy for systematic examination. Additionally, although foundational studies on m6A in pregnancy-associated processes have demonstrated translational potential, they still lack clinical validation, and the pathway from mechanistic discovery to clinical application remains unclear.

This review aimed to systematically summarise the current knowledge on the roles of m6A in both physiological and pathological contexts of pregnancy, critically synthesized the available evidence, and attempted to elucidate the potential mechanisms by which the dynamic regulatory network of m6A may orchestrate the coordinated regulation of the maternal-foetal immune microenvironment, placental function, and foetal development. Building on this foundation, we further explored the potential and challenges associated with translating m6A-related molecules into clinical diagnostic biomarkers or therapeutic targets. Our goal was to establish a framework for understanding the impact of m6A on women’s reproductive health, ultimately contributing to innovative diagnostic and treatment strategies targeting the m6A pathway.

## 2 m6A regulators

Among the various types of RNA modification, m6A modification is the most common and abundant type of posttranscriptional modification in eukaryotes and is characterised by its dynamism and reversibility (2). This dynamic process is coordinated by three specific types of proteins: writing proteins, erasing proteins and reading proteins. They jointly regulate key aspects of RNA metabolism, such as selective splicing, transport, stability and translation. Advances in detection technologies have revealed that m6A modification affects more than 7,000 mRNA transcripts in the human transcriptome, as well as a variety of other RNA molecules, such as lncRNAs, miRNAs, and circRNAs (13–15). Furthermore, m6A modification is highly conserved, is significantly enriched within the RRACH sequence motif (where R denotes A/G and H denotes A/C/U), and is predominantly distributed in coding sequences, the 3’ untranslated region, around stop codons, and within long exonic regions (13).

### 2.1 Writer

m6A writers are defined as a class of methyltransferases that deposit methyl groups onto the N6 position of adenosine residues in target RNAs, utilising S-adenosylmethionine (SAM) as a cofactor. Their core catalytic function is typically executed by the methyltransferase complex (MTC), which ensures the precision and efficiency of the methylation process (16, 17). The m6A MTC comprises a catalytic METTL3–METTL14 heterodimer and several regulatory subunits, including WTAP, VIRMA (KIAA1429), RBM15/RBM15B, and ZC3H13.

METTL3 is the first m6A methyltransferase to be discovered. As the main catalytic subunit, it uses its highly active methyltransferase domain to promote methyl transfer (18, 19). Although METTL14 lacks catalytic activity, it acts as a key metamorphic activator and structural support. METTL14 and METTL3 are located in nuclear spots, which not only stabilizes the structure of the MTC but also identifies specific RNA sequence sequences through its unique RNA binding domain, thus guiding the complex to the precise substrate RNA position (18, 20). As a connecting protein, WTAP recruits METTL3-METTL14 isodimers to nuclear spots, thus depositing m6A in a specific RNA region (21).

ZC3H13 anchors MTC in the nuclear spot in a coordinated way and establishes a spatial framework to promote site-specific m6A methylation (22, 23). In addition, VIRMA and RBM15 guide MTC to specific RNA sites, including areas near 3’ UTR and termination codons, to maintain the m6A level in these regions (16, 24).

**Figure 1:**
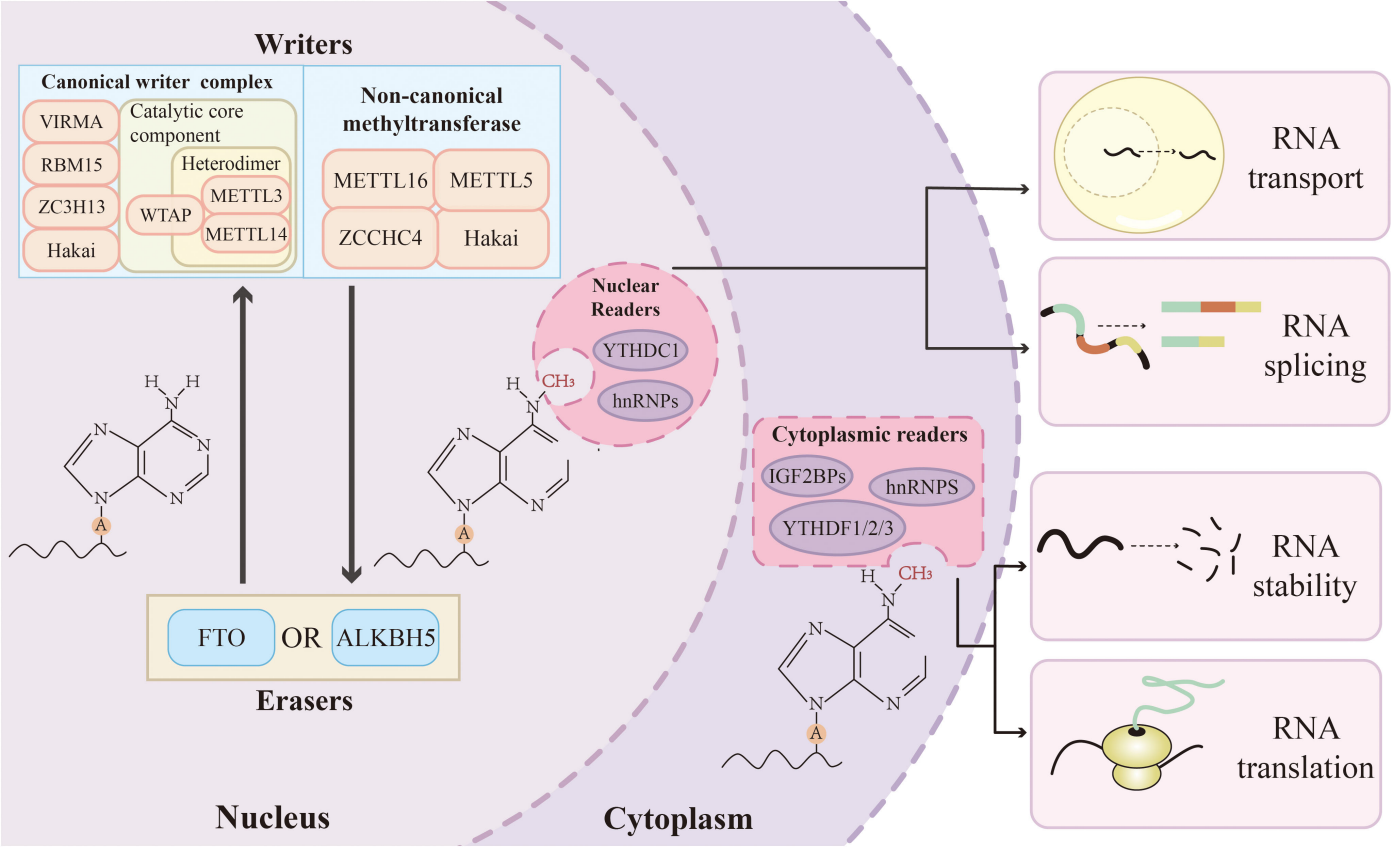
The mechanism of m6A modification. The m6A modification is dynamically regulated by writer, eraser, and reader. Writers catalyse the methylation of adenosine residues. Erasers remove this modification. Readers, including proteins from the YTH domain protein, IGF2BP, and hnRNP families, recognize the m6A modification and recruit effector proteins, thereby modulating RNA metabolism, including transport, splicing, stability, and translation.

In addition to the classic MTC, ZCCHC4, METTL16, METTL5, and Hakai have been identified as new methyltransferases. Hakai has recently been confirmed to be a component of MTC in fruit flies and human cells. It plays a key role in stabilising MTC through interactions with WTAP and ZC3H13. Although its potential ubiquitination-related functions require further validation, these findings suggest a previously unrecognised mechanism of crosstalk between epitranscriptomic modification and posttranslational regulation (25). METTL16 primarily catalyses m6A methylation on specific mRNAs (e.g., MAT2A transcripts) and noncoding RNAs (such as U6 snRNA) (26, 27). Through its role in dynamically regulating the methylation of target RNAs, this enzyme is involved in critical biological processes, such as maintaining intracellular SAM homeostasis and RNA splicing. In addition, METTL5 and ZCCHC4 are recognised as m6A methyltransferases that function independently of MTC and modify 18S and 28S ribosomal RNAs, respectively (28, 29) (**Figure 1**).

### 2.2 Eraser

The reversibility of m6A modification is ensured by dedicated erasers, which are demethylases that catalyse the demethylation of N6-methyladenosine (30, 31). The two well-established m6A erasers are FTO and ALKBH5, both of which are part of the ALKB family of dioxygenases that require Fe(II) and α-ketoglutarate as essential cofactors for their catalytic activity (32, 33).

The demethylation of m6A catalysed by FTO occurs through sequential oxidative reactions within its catalytic centre (34). Notably, FTO regulates the splicing of precursor mRNA by removing m6A modification; otherwise, m6A modification hinders the binding of the splicing factor SRSF2 to its target transcript, thus finely regulating the selection of the splicing site (14). Similarly, ALKBH5 is located mainly in the nucleus and enriched in nuclear spots. It directly oxidises m6A to restore unmodified adenosine (35). Its nuclear positioning enables it to effectively target the new transcription precursor mRNA, and by dynamically altering its m6A level, ALKBH5 directly affects nuclear output, metabolic stability and transcript processing (30, 36, 37) (**Figure 1**).

In addition to these core RNA-erasing enzymes, new evidence shows that the protein family has a broader substrate spectrum. Recent research progress shows that ALKBH3 also has catalytic activity on m6A modification in tRNA, which broadens people’s understanding of its RNA modification function. However, the detailed molecular mechanism behind this activity has yet to be further experimentally verified (38).

### 2.3 Reader

m6A-reading proteins are a class of effectoproteins that specifically bind to m6A-modified RNA, thus affecting its metabolism and function. Currently known m6A-reading proteins include mainly the YTH domain protein family, the IGF2BP protein family, the hnRNP protein family, and eIF3 (39, 40). On the basis of their main subcellular localisation and functional background, these proteins can be roughly divided into intranuclear reading proteins and cytoplasmic reading proteins, but some members, such as YTHDC2, exhibit mixed localisation.

Intranuclear readers are mainly involved in the processes of precursor mRNA selective splicing, structural conversion, and nucleoplasm output. The nuclear-specific YTHDC1 regulates the exon inclusion by recruiting the splicing factors SRSF3 and SRSF10, thus playing the key regulatory role of selective splicing (41). It also promotes the nuclear export of m6A-modified mRNAs through interactions with SRSF3 and the export receptor NXF1 (42). Another YTH family member, YTHDC2, shuttles between the nucleus and cytoplasm and demonstrates context-dependent roles in enhancing both the translation and degradation of its target mRNAs (43, 44). The hnRNP famil**y** (e.g., HNRNPC, HNRNPG, HNRNPA2B1) are predominantly nuclear readers and engage in alternative splicing and transcript processing via their RRM domains (45). Among them, HNRNPC and HNRNPG, as nuclear RNA-binding proteins, have binding activities that can be regulated by RNA structural changes induced by m6A modification, thereby influencing pre-mRNA abundance and splicing patterns (45, 46). HNRNPA2B1 can recognize m6A modifications and subsequently recruit the DGCR8 complex to promote primary microRNA processing (47).

Cytoplasmic readers primarily regulate mRNA translation and stability. YTHDF1 can improve the translation efficiency of m6A-modified mRNA (48). YTHDF2 regulates the degradation of m6A-modified mRNA through the collection and initiation of de-adenosine acidification mediated by the CCR4-NOT complex (49). YTHDF3 and YTHDF1 have significant binding site overlap and cooperate to promote the translation of common target mRNAs. In contrast, YTHDF3 also interacts with YTHDF2 to promote the degradation of mRNA (50, 51). However, the recently proposed unified model shows that YTHDF1/2/3 are functionally redundant and promote the degradation of m6A-modified mRNA through phase separation, thus reducing the overall expression level of the target mRNA. This model is supported by functional experiments; that is, only by knocking down these three collateral homologous genes at the same time (rather than by knocking down them alone) can the target mRNA significantly stabilise (52).

The IGF2BP family (IGF2BP1/2/3) is characterised by m6A modifications through its KH and RGG domains and specifically binds to target mRNAs and then recruits RNA stabilisation factors to increase mRNA stability (53). In addition, m6A-dependent identification of the 3’UTR of IGF2BP can stabilise the interaction between ribosomes and mRNAs in polyribosomes, thus improving the accuracy of translation (54, 55). This double regulation of mRNA stability and translation highlights the important role of IGF2BP in the dynamic regulation of gene expression. In addition, evidence has shown that m6A in the 5’UTR can directly recruit the translation-initiating factor eIF3, thus promoting translation that does not depend on the cap structure and expanding the mechanical role of m6A in translation regulation (56) (**Figure 1**).

### 2.4 Distribution characteristics of m6A

The distribution of m6A on RNA transcripts is not random but shows a highly specific and evolutionarily conserved pattern, which forms the basis for its role in the gene regulation network. In mRNA, m6A modification shows significant sequence specificity, which occurs mainly in the RRACH cosequence sequence (R = G or A; H = A, C or U). In terms of spatial distribution, m6A shows obvious regional preference, with significant enrichment near the transcription start site (TSS), in the coding sequence (CDS) and in the 3’ nontranslated region (3’UTR). Notably, the highest density of m6A usually occurs around the termination codon of the CDS and in the near-quarter region of the 5’ end of the 3’UTR. From mice to humans, the specificity pattern modified by m6A is highly conserved, highlighting its functional significance (57–59). Given the key role of the 3’UTR as a miRNA binding platform, the enrichment of m6A in this region indicates that it may participate in regulating miRNA-mediated gene regulation (60).

In addition to mRNA, m6A modifications are also common in a variety of non-coding RNAs, including tRNA, rRNA, snRNA, snoRNA, and lncRNA (61). These modifications play a crucial role in maintaining the structural integrity of non-coding RNA and regulating its biological function. A typical example is the primary miRNA (pri-miRNA) transcript, whose m6A modification is significantly enriched. The m6A marker on pri-miRNA can be specifically identified by the reading protein HNRNPA2B1, and HNRNPA2B1 in turn collects the DGCR8 complex to promote the processing and maturation of pri-miRNA (47).

In addition, the global abundance and transcription book-specific distribution patterns of m6A are not uniformly distributed but significantly differ across different tissues and cell types. For example, the overall m6A levels of tissues such as the brain, liver and testicles are high, and the modified transcripts in each tissue differ (62). Increasing evidence has shown that the m6A modification spectrum is highly tissue specific and closely related to the unique biological functions of different organs. For example, a multiorgan sequencing study revealed that the placenta is particularly rich in m6A modification (63). Comparison of the m6A maps of foetal and adult tissues further revealed that m6A modification in foetal tissue is preferentially enriched in the coding sequence (CDS). This finding is in stark contrast to the typical distribution of m6A in adult tissues, which is distributed mainly near the termination codon and within the 3’UTR (64). This unique distribution strongly suggests that m6A plays a unique regulatory role in the dynamic process of embryonic development.

## 3 Detection techniques for m6A modification

The emergence of m6A sequencing technology has advanced the field of epitranscriptomics. Currently, multiple m6A detection techniques have been developed, each with distinct characteristics to meet detection demands across different research scenarios (**Table 1**). Using these sequencing approaches, researchers can map m6A modification profiles across the transcriptome, perform stoichiometric analysis, and delve into its functional mechanisms. Based on their underlying principles and resolution, current detection methods can be broadly categorized into several types.

**Table 1:** Comparison of mainstream m6A detection technologies.

Technology	Resolution	Sensitivity	Characteristic	Applicability	Reference
MeRIP-seq/m6A-seq	100-200nt	Medium	Stable and convenient;however, limited by low resolution and potential antibody cross-reactivity.	Suitable for preliminary large-scale profiling of m6A transcriptomes	(1)
miCLIP-seq	Single-nucleotide	High	Enables single-base resolution but involves complex procedures and high antibody dependency, which may lead to elevated background noise.	Single-base resolution m6A site detection	(2)
m6A-SEAL	Bulk	High	Time- and cost-efficient, antibody-independent; detection efficiency is influenced by enzymatic activity and labeling performance.	Suitable for rapid and low-cost enrichment of m6A sites without antibodies.	(3)
m6A-SAC -seq	Single-nucleotide	High	Antibody-free and requires low RNA input; however, the procedure is complex and enzymes may exhibit sequence bias.	Whole-transcriptome m6A mapping/minuscule samples	(4)
DART-seq	Single-nucleotide	Low	Dependent on cell transfection; overexpression artifacts may occur.	Samples with extremely low starting RNA quantities	(5)
MAZTER-seq	Single-nucleotide	High	Antibody-independent but restricted to ACA motifs and offers limited detection coverage.	Detection and quantification of m6A sites in ACA motifs	(6)
m6A-REF	Single-nucleotide	High	Antibody-free with ACA motif specificity.	Enables detection and quantification of m6A at ACA sites	(7)
eTAM-seq	Single-nucleotide	High	No sequence preference, low RNA input required; results are sensitive to enzyme quality.	Quantitative analysis of m6A in low-volume samples	(8)
DRS	Single-nucleotide	Medium	Direct sequencing preserves native RNA modifications; data interpretation remains complex.	Transcript isomer-specific m6A analysis/single-molecule level m6A heterogeneity study	(9, 10)
GLORI	Single-nucleotide	High	Stable and reproducible; however, chemical treatment may compromise RNA integrity.	Enables robust m6A quantification at single-base resolution	(11)
scm6A-seq	Single-cell	High	Allows identification of rare cell types via m6A signatures; technically demanding and operationally challenging.	Rare cell types/early embryonic development research	(12)
LC-MS	N/A	High	Accurate and sensitive for absolute quantification but lacks sequence and locus information.	precise measurement of global m6A levels	(13, 14)
m6A-ELISA	N/A	Low	Simple and rapid; cannot identify specific m6A sites.	rapid comparison of global m6A levels in clinical settings.	(15)

### 3.1 m6A-seq based on antibody-dependent techniques

**Figure 2:**
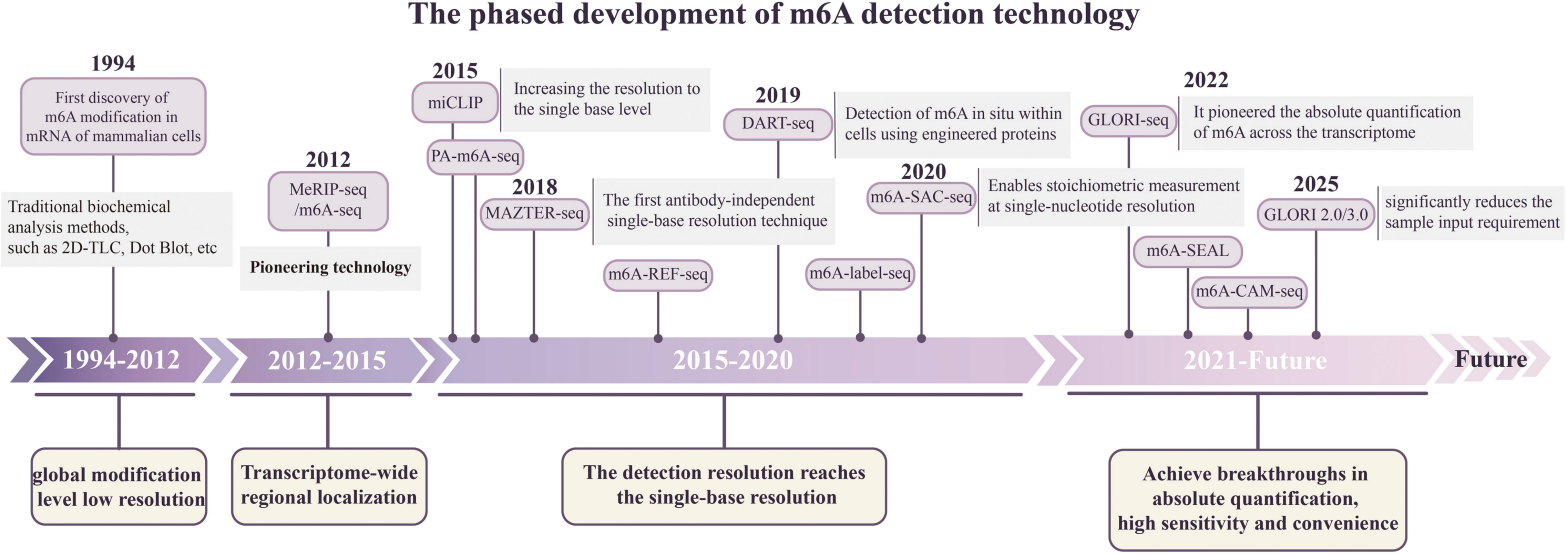
The phased development of m6A detection technology. MeRIP-seq provided the first transcriptome-wide maps of m6A, albeit with limited resolution. Crosslinking-assisted techniques such as miCLIP achieve single-base resolution. Enzyme-based, chemistry-based, and domain-based protein strategies offer alternative, antibody-independent routes for m6A detection. Emerging technologies (e.g., GLORI-seq) have further enabled absolute quantitative analysis of m6A modifications.

Pioneering MeRIP-seq/m6A-seq technology was established in 2012; m6A-specific antibodies were used to immunoprecipitate methylated RNA fragments, after which high-throughput sequencing was performed (13). This method can identify the m6A enrichment region with a resolution of 100–200 nt and reveal the characteristic accumulation of m6A near the termination codon and within the 3’UTR (59) (**Figure 2**). Although MeRIP-seq/m6A-seq is widely used, it cannot distinguish between m6A and m6Am, and its resolution is limited (65).

MeRIP-seq/m6A-seq has been widely applied for whole-transcriptome m6A profiling in pregnancy-related tissues, including the placenta andfoetalorgans. For instance, integrated analysis using MeRIP-seq and mRNA-seq revealed genome-wide distribution patterns of m6A modifications in foetal livers from mice with GDM (66). Wang et al. (67) performed MeRIP-seq on placentas from preeclampsia (PE) and control groups, identifying 685 differentially methylated m6A peaks predominantly enriched in the 3′UTR and CDS regions. Their findings provide a crucial foundation for elucidating how m6A contributes to PE pathogenesis by modulating mRNA stability and translation.

To improve the accuracy of sequencing, an m6A sequencing strategy assisted by optical cross-linking has been developed. At present, the commonly used representative technologies include miCLIP and PA-m6A-seq. miCLIP uses 254-nm ultraviolet light to cross-link m6A antibodies with RNA to form a covalent complex. In the subsequent reverse transcription process, these sites produce characteristic mismatches and termination signals to achieve accurate identification of m6A with single-base resolution (68) (**Figure 2**). The PA-m6A-seq scheme combines antibody-based cross-linking and 4-thiourin (4SU) labelling, which can achieve precise positioning of m6A. Covalent crosslinking at 365 nm is followed by RNA fragmentation to 30 nt, and the 4SU-derived T-to-C transitions provide nucleotide-level landmarks, achieving a final mapping resolution of approximately 23 nt (69).

### 3.2 m6A-seq based on enzyme-assisted or chemical labelling

Antibody-dependent m6A sequencing techniques suffer from limitations such as nonspecific binding and sequence bias. To address these limitations, enzyme-based detection techniques have begun to emerge. MAZTER-seq (70) and m6A-REF-seq (71) leverage the bacterial endonuclease MazF, which cleaves unmodified ACA motifs but leaves m6A-modified ACA sites intact, allowing for base-resolution mapping and quantification of m6A within ACA contexts (**Figure 2**). However, the MazF endonuclease in MAZTER-seq targets only the ACA motif, which accounts for approximately 16% of the classic DRACH m6A consensus sequence. Consequently, MAZTER-seq cannot locate all m6A modification sites (72).

m6A-label-seq is a metabolic labelling method that maps transcriptome-wide m6A modifications at single-base resolution. This technique replaces m6A with a6A and utilises mismatches generated by a6A during reverse transcription to identify m6A sites (73). Additionally, m6A-SAC-seq is a selective allylation chemical labelling-based technology that can map transcriptome-wide m6A at single-nucleotide resolution while also yielding stoichiometric information (74).

### 3.3 m6A-seq based on m6A proteins

DART-seq employs an APOBEC1-YTH fusion protein that catalyses C-to-U deamination adjacent to m6A sites, enabling detection through standard RNA-seq (75). While it enables cell type-specific m6A profiling, its application in tissue samples is limited by transfection efficiency, and the YTH domain recognises approximately 60% of m6A sites (**Figure 2**).

m6A-SEAL, an FTO-assisted chemical labelling m6A sequencing method, involves FTO-mediated oxidation of m6A to hm6A, subsequent conversion to dm6A through disulfide-mediated thiol addition, and, ultimately, biotinylation (76). Compared with alternative m6A sequencing and specific validation techniques, the key advantages of m6A-SEAL are its high sensitivity and specificity for the consistent detection of transcriptome-wide m6A modifications.

### 3.4 Emerging single-cell m6A-seq technologies

GLORI-seq achieves absolute m6A quantification through glyoxal and nitrite-mediated deamination, where unmodified adenosines are converted to inosines [detected as adenine (A) to guanine (G) mutations], while m6A residues remain unchanged (77). Notably, to address the issues of severe RNA degradation and high sample input requirements in the original workflow, the technical team optimised and developed GLORI 2.0 and GLORI 3.0. These improved versions employ a simplified one-pot deamination reaction, which is gentler and faster. This method effectively maintains the integrity of RNA while maintaining a high A-to-I conversion efficiency and significantly reducing the amount of required RNA input (78). Although GLORI-seq has not yet been fully achieved as a single cell, its single-base resolution and absolute quantification ability have laid the foundation for the future expansion of single cells.

Dynamic regulation of m6A modification is crucial during mammalian oocyte and early embryonic development. However, conventional MeRIP-seq is not applicable due to the extremely low sample input. To address this limitation, Yang et al. (79) developed scm6A-seq by integrating RNA multiplex labelling with MeRIP-seq, enabling m6A sequencing in single cells or ultra-low-input embryonic samples. This technique, leveraging its high resolution, systematically delineated for the first time the dynamic patterns of m6A modification during oocyte maturation and early embryonic development. Furthermore, it revealed epitranscriptomic heterogeneity among individual blastomeres. This heterogeneity is closely associated with asynchrony in zygotic genome activation (ZGA), suggesting that m6A may participate in early cell fate determination by modifying key transcription factor mRNAs (79). Further studies demonstrated that m6A modification levels are positively correlated with translational efficiency during oocyte maturation. Building on this, the research team also developed the C2T-APP analysis pipeline, which enables simultaneous profiling of m6A, m6Am, m7G cap structures, and poly(A) tail lengths (80).

Dynamic regulation of m6A modification is crucial during mammalian oocyte and early embryonic development. However, conventional MeRIP-seq is not applicable due to the extremely low sample input. To address this limitation, Yang et al. developed scm6A-seq by integrating RNA multiplex labelling with MeRIP-seq, enabling m6A sequencing in single cells or ultra-low-input embryonic samples (79). Leveraging its high resolution, this technique systematically delineated, for the first time, the dynamic patterns of m6A modification during oocyte maturation and early embryonic development and revealed epitranscriptomic heterogeneity among individual blastomeres. This heterogeneity is closely associated with asynchrony in ZGA, suggesting that m6A may participate in early cell fate determination by modifying key transcription factor mRNAs (79). Further studies demonstrated that m6A modification levels are positively correlated with translational efficiency during oocyte maturation. Building on this, the research team also developed the C2T-APP analysis pipeline, which enables simultaneous profiling of m6A, m6Am, m7G cap structures, and poly(A) tail lengths (80).

In addition to the abovementioned classic m6A detection technology, direct RNA sequencing (DRS) is unique. This technology directly reads sequences by measuring the change in ion current through complete RNA molecules through nanopores (**Figure 2**). During this process, m6A modification causes characteristic changes in electrical signals (81). This technology itself cannot achieve single-cell sequencing. It needs to be combined with relevant deep learning algorithms and single-cell separation techniques to reach single-cell resolution (82). Deep learning algorithms, represented by m6Aiso, combined with endogenous labelling and DRS, have for the first time enabled the accurate identification and quantification of m6A modification sites on individual intact RNA molecules, revealing the differential m6A modification at the same site across distinct isoforms and its underlying regulatory mechanisms (83).

### 3.5 Other m6A-seq

High-performance liquid chromatography (HPLC) is separated on the basis of differences in nucleotide polarity to achieve rapid detection and quantification of RNA modifications. However, owing to the limited sensitivity of ultraviolet spectrophotometers, HPLC is usually only suitable for the study of high-abundance modifications (84).

Liquid chromatography–mass spectrometry (LC–MS) was developed on this basis; although it also uses HPLC for separation, it replaces ultraviolet detectors with a high-sensitivity mass spectrometer for nucleotide identification (85). An increase in this core detector significantly improves the overall sensitivity of the method.

LC-MS, owing to its precise quantitative capability, is widely employed for the detection and analysis of global m6A level alterations. For example, by measuring m6A modification levels in uterine tissues at different stages of mouse pregnancy and calculating the m6A/A molar ratio to achieve absolute quantification, studies have demonstrated that uterine m6A levels progressively increase throughout gestation. This global and quantitative trend has laid an essential foundation for subsequent research (86).

## 4 m6A in oogenesis

Oogenesis is a complex and dynamic process that spans from the embryonic stage to postpuberty (87). This process begins during embryonic development, when the primitive germ cells in the foetal ovaries change from undergoing continuous mitosis to undergoing meiosis. This transformation eventually causes the oocytes to stagnate during the double-line period of premeiosis I (MPI), that is, the foaming (GV) period, and form primitive follicles with the surrounding monolayer of flat granular cells (88). After entering puberty, the follicle activation process is activated under the regulation of periodic gonadotropins. This stage is characterised by oocyte growth and granulocyte proliferation, in which the selective mechanism dependent on follicle stimulation (FSH) promotes the maturity of dominant follicles (89). Under the triggering of peak luteinising hormone (LH) levels before ovulation, primary oocytes stagnant during the GV period resumed meiosis. This recovery process is marked by foaming rupture (GVBD), first pole discharge, and subsequent stagnation in meiosis II (MII), where the egg stagnates until fertilisation (90). Throughout the process, maternal mRNA is synthesised and accumulates in large quantities during the growth stage of oocytes. However, transcriptional activity is largely silenced in fully grown GV-stage oocytes, which is critical for oocyte maturation. m6A modification orchestrates key events during oogenesis by controlling maternal mRNA metabolism and mediating posttranscriptional regulation, such as alternative splicing, degradation, and translation.

### 4.1 Role of m6A in oocyte meiosis

Proper initiation and arrest of the first meiotic division are crucial prerequisites for the successful transition from mitosis to meiosis. YTHDC2 plays key roles in the initiation and maintenance of prophase during oocyte meiosis. Studies have shown that in foetal wild-type mouse ovaries, the expression of YTHDC2 is significantly upregulated, and YTHDC2 colocalises with the meiotic marker protein SYCP3. Conditional knockout of YTHDC2 in female mice resulted in ovarian hypoplasia and a failure to establish the primordial follicle pool. The underlying cause is the inability of mutant germ cells to properly initiate the meiotic program, manifested as aberrant chromosome condensation and persistent expression of mitotic cyclins (e.g., Cyclin D1). These findings demonstrated that YTHDC2 is a key molecule responsible for terminating mitosis and initiating meiosis (43).

Further research revealed that YTHDC2 forms a functional complex with MEIOC, which recognises and degrades a set of mitosis-related mRNAs, thereby actively suppressing the return of germ cells to the mitotic program and ensuring the maintenance of prophase I of meiosis (91). These findings suggest that the YTHDC2-MEIOC complex may employ a similar mRNA stability regulatory mechanism to maintain meiotic arrest in oocytes.

The m6A regulatory network is equally critical for the resumption and maturation of meiosis. Oocyte meiotic resumption is marked by GVBD, a process highly dependent on the precise polyadenylation and translation of stored maternal mRNAs. YTHDC1, an important nuclear m6A reader, is central to this process. It is strongly expressed in postnatal oocytes, MII-stage eggs, and preimplantation embryos, but its expression level is relatively low in GV-stage oocytes. Conditional knockout studies have shown that loss of YTHDC1 significantly diminishes GV-stage oocytes in mutants, and the surviving oocytes fail to resume meiosis (41).

Another m6A reader, YTHDF3, is also essential for meiotic resumption in oocytes. Research indicates that YTHDF3 promotes the translation of specific maternal transcripts by recognizing m6A modifications. Knockout of YTHDF3 results in a global decrease in translation efficiency, affecting meiosis-related genes (such as those involved in chromosome segregation and DNA repair), which consequently suppresses first polar body extrusion in mouse oocytes (92). It is worth noting that the m6A modifier is also crucial to the recovery and maturation of oocyte meiosis. For example, KIAA1429 is significantly upregulated in oocytes, and its absence will cause a significant decrease in the level of m6A in GV oocytes, which will lead to developmental stagnation (93).

**Figure 3:**
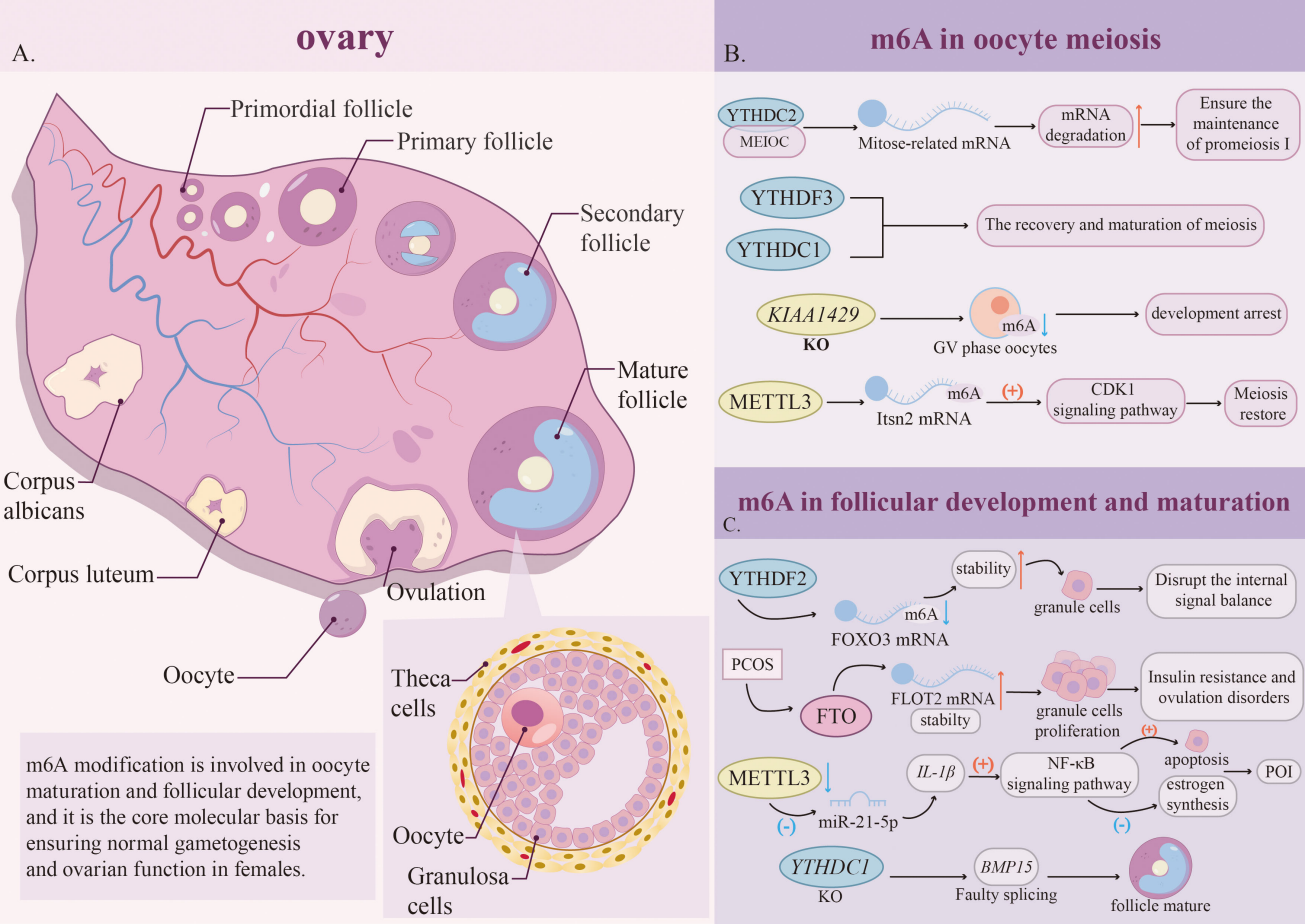
The dynamic functional roles of m6A RNA methylation in oogenesis. **(A)** The follicular development cycle begins with primordial follicles, progressing through the primary follicular phase, secondary follicular phase, and mature follicular phase, ultimately culminating in ovulation and corpus luteum formation. m6A modification plays a central role in this process, supporting normal oocyte maturation and follicular development. **(B)** m6A modification precisely regulates oocyte meiosis through a dynamically regulated network comprising writer, reader, and eraser. **(C)** m6A plays a crucial role in follicular development and maturation, and its dysregulation is closely associated with diseases such as PCOS and POI. PCOS, Polycystic ovary syndrome; POI, Primary ovarian insufficiency.

More direct evidence comes from the study of the core methyltransferase METTL3, which promotes the recovery of meiosis by enhancing the stability and translation activity of Itsn2 mRNA, thus activating the CDK1 signalling pathway. In contrast, the absence of METTL3 can damage the synthesis of the ITSN2 protein, destroy CDK1 signalling, lead to spindle assembly defects, and eventually lead to GVBD failure and stagnation of meiosis in phase I (94). Recent studies have revealed the key role of the demethylase ALKBH5 in oocyte meiosis: specific knockout of the ALKBH5 gene prevents oocytes from normally completing meiosis phase I (MI) after GVBD and significantly reduces the discharge rate of the first pole (35) (**Figure 3A**). Taken together, these findings emphasise the importance of dynamic m6A regulatory networks in the normal progression of oocyte meiosis.

### 4.2 Role of m6A in follicular development and maturation

Granular cells (GCs) are the core cell components of follicles and directly regulate the development of oocytes through paracrine signalling and physical contact. m6A modification plays a key role in maintaining the normal development and maturity of follicles by precisely regulating the gene expression network in GCs. Disorder of the m6A network is an important mechanism of GC dysfunction and related diseases. In normal GCs, YTHDF2 recognises m6A modifications in the 3’-UTR of FOXO3 mRNA, thereby promoting its degradation. However, in the GCs of patients with PCOS, the m6A modification of FOXO3 mRNA is reduced, and its expression is no longer regulated by m6A methyltransferase or demethylase. This obstacle to m6A recognition leads to abnormal stability of FOXO3 mRNA, which in turn destroys the signal balance in GCs (95).

Another study indicated that FTO regulates GC function by upregulating FLOT2. In patients with PCOS, overexpressed FTO in GC will eliminate m6A modification on FLOT2 mRNA, thus enhancing its stability and expression. This then promotes GC proliferation and impairs the translocation of glucose transport protein 4 (GLUT4). It eventually leads to insulin resistance and ovulation disorders, and lowering FLOT2 can alleviate these changes. These two studies together provided an transcriptomic explanation for the observed follicle development stagnation in PCOS patients.

In the context of POI and related ovarian dysfunction, studies show that the downward regulation of METTL3 will lead to a decrease in m6A modification on pri-miR-21, thus inhibiting the production of mature miR-21-5p. This leads to the upregulation of its target gene IL-1β. Subsequently, the activation of the NF-κB-mediated signalling pathway driven by IL-1β will trigger GC cell apoptosis, inhibit autophagy, and reduce oestrogen synthesis, resulting in stagnation of follicular development (96, 97). This highlights the key role of METTL3 in maintaining the follicular microenvironment.

Under physiological conditions, the m6A regulatory mechanism is crucial for ensuring the normal development of follicles. The nuclear reader YTHDC1 influences secondary follicle formation by regulating alternative precursor mRNA polyadenylation (APA) and splicing. Its deficiency causes aberrant splicing of key folliculogenic genes, such as BMP15, thereby impairing follicular maturation (41). Another reading protein, YTHDC2, is also essential for female fertility. Its specific absence can directly lead to ovarian atrophy, stagnation of germ cell development, and loss of fertility (98, 99) (**Figure 3B**). These findings confirmed the fundamental role of the m6A regulatory network in follicular development.

## 5 m6A modification in early embryonic development

**Figure 4:**
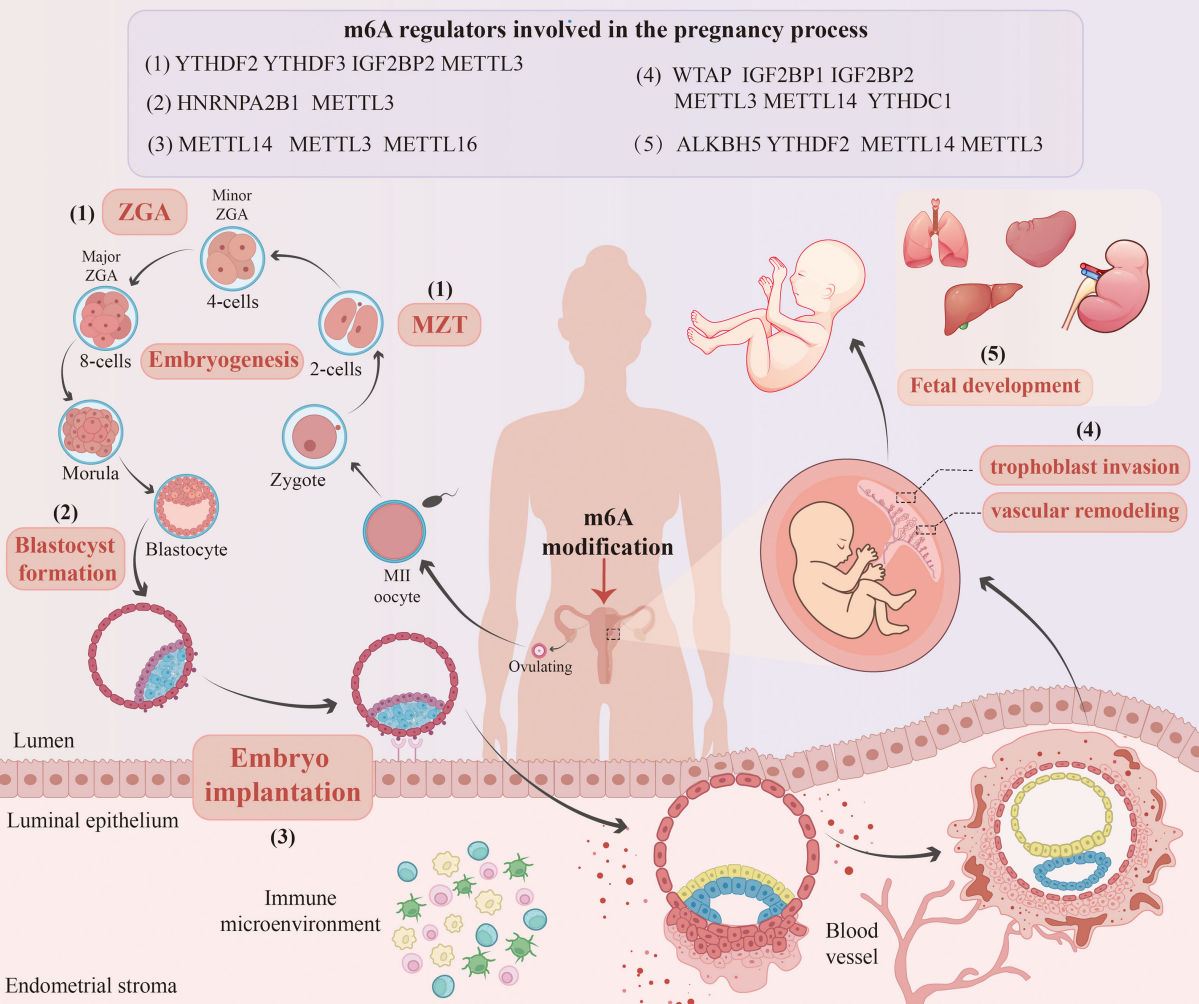
Overview of m6A modifications involved in the pregnancy process. Successful pregnancy relies on a series of precisely coordinated physiological processes, in which m6A modification plays a critical role throughout multiple key stages. From early events including ZGA, blastocyst development, embryo implantation, and the establishment of endometrial receptivity, to subsequent processes such as trophoblast cell invasion, spiral artery remodeling, and foetal development, m6A modification exerts essential regulatory functions. When this epitranscriptomic regulatory network becomes dysregulated, any of these processes may be disrupted, leading to abnormal embryonic development or even pregnancy failure.

m6A modification is a key epitome regulatory factor in early embryonic development. Its regulation involves a number of important events, including maternal–zygotic transformation (MZT), blastocyst formation, embryo implantation, and endometrial tolerance. In this process, the dynamic regulatory network, which is composed of m6A write enzymes, erase enzymes and read enzymes, ensures the orderly development of early embryos. It achieves this goal by precisely regulating key events, including maternal mRNA degradation, ZGA, blastocyst formation, and endometrial receptivity (**Figure 4**). Studies of functional deletion have clearly confirmed that the absence of core m6A regulatory components can directly cause serious embryonic developmental defects, including embryonic stagnation. In summary, m6A modification constitutes a multifaceted and accurate posttranscriptional regulation system that is crucial to the success of early embryonic development.

### 5.1 Role of m6A in regulating MZT and ZGA

MZT marks a key turning point in early embryonic development, which includes the activation of ZGA and the procedural elimination of maternal mRNA (100). Disorders of MZT or ZGA can lead to embryonic development stagnation and pregnancy failure (101, 102). m6A modification is a key regulatory factor in this biological process.

m6A-mediated maternal mRNA degradation is a key mechanism to ensure the normal transformation of MZT. He et al. (103) used the zebrafish model to prove that YTHDF2 is a key factor driving MZT by identifying the m6A marker on the mother source transcript and promoting its degradation. Subsequently, studies in goat embryos confirmed that knocking down YTHDF2 will destroy the removal of maternal mRNA, thus delaying ZGA and leading to goat embryo developmental defects (104). These studies established the core role of YTHDF2 in the removal of m6A-dependent maternal mRNA.

It is noteworthy that the understanding of this model is continuously evolving. Kontur et al. (105) revealed a functional redundancy between YTHDF2 and YTHDF3. While individual knockouts showed no effect on maternal mRNA decay or ZGA, their combined loss caused profound MZT defects and compromised early embryogenesis. This study suggested that m6A-mediated regulation of maternal mRNA fate may not rely solely on a single reader protein but is likely achieved through functional redundancy and cooperation among YTHDF family proteins, with potential species-specific differences in the underlying mechanisms.

Furthermore, other types of m6A regulatory proteins are involved in MZT. For instance, the reader protein IGF2BP2, which stabilises specific transcripts such as Ccar1 and Rps14, is essential for maintaining the developmental potential of mouse embryos. Maternal deficiency of IGF2BP2 results in developmental arrest at the 2-cell stage (106). In addition, the m6A writer complex is indispensable for MZT. Research has shown that oocyte-specific knockout of METTL3 disrupts the maternal mRNA decay program, inhibits meiotic maturation, perturbs the initiation of ZGA, and ultimately causes developmental arrest at the MZT stage (107).

### 5.2 Role of m6A in blastocyst formation

Blastocyst formation relies on precise gene expression regulation to coordinate cell proliferation, differentiation, and morphogenesis. Evidence shows that m6A modification plays a central role in this process by regulating key processes such as multienergy networks and cell cycle processes.

As an m6A reading protein, HNRNPA2B1 is crucial for maintaining the characteristics of the cell mass in the blastocyst. Functional studies have confirmed that knocking down HNRNPA2B1 expression slows the growth of mouse embryos after the 4th cell cycle and significantly reduces the rate of blastocyst formation. The resulting blastocysts are small in size and poor in quality and cannot form clones from normal internal cell masses (ICMs) outside the body (108). This phenotype mirrors mechanisms in human embryonic stem cells (hESCs), where HNRNPA2B1 depletion disrupts pluripotency maintenance by suppressing OCT4/NANOG/SOX2, accelerates epithelial–mesenchymal transition (EMT), and induces G0/G1 arrest through the concerted downregulation of Cyclin D1, Cyclin E, and Cdc25A (109). These findings reveal the molecular basis through which HNRNPA2B1 is involved in both the maintenance of pluripotency and cell cycle processes to ensure normal intracellular development.

In addition, the steady state of METTL3 expression is crucial to the development of blastocysts. Gao et al. (110) reported that knockdown or overexpression of METTL3 significantly reduced the rate of blastocyst development and the number of trophic ectoderm (TE) cells. More importantly, METTL3 strongly promotes the phase separation of the RNA-binding protein Rbm14 through its catalytic m6A modification activity, thus promoting the formation of biomolecular condensates and enhancing its binding ability with the parent mRNA. In contrast, m5C modification inhibits this process (111). This antagonistic effect between RNA modifications ensures the spatial and temporal accuracy of mRNA metabolism and guarantees normal development of the blastocyst.

### 5.3 Role of m6A in embryo implantation

Embryo implantation is a crucial developmental phase during which the blastocyst forms a structural connection with the maternal uterine wall to facilitate material exchange. Research has shown that m6A methyltransferases, including the METTL3/METTL14 complex and METTL16, are crucial for the success of this process through the regulation of gene expression and cell fate decisions.

Endometrial tolerance is precisely regulated by the ovarian steroid hormones oestrogen and progesterone. METTL14 has been proven to be a key factor in this process, because knocking out the uterine-specific METTL14 gene in mice will lead to oestrogen receptor α (ERα) signalling pathway disorder, manifested as abnormal pathway upregulation and receptor phosphorylation. This signalling pathway disorder is accompanied by immune cell infiltration, which will eventually damage embryo implantation (112). Knocking out METTL3 or METTL14 in the embryonic stem cell line will also lead to a general decrease in the level of m6A, which in turn leads to impaired differentiation and post-implantation developmental stagnation, which confirms that the METTL3/METTL14 complex plays a crucial role in maintaining the cell potential required for implantation (113).

Evidence confirms that METTL16 plays a crucial role in embryo implantation. Studies have shown that knocking out the METTL16 gene in mouse embryos decreases the expression level of MAT2A mRNA, thus inducing SAM metabolic disorders. This leads to large-scale transcriptome dysplasia in the blastocyst, hindering normal embryonic development and causing developmental stagnation before and after implantation (114).

### 5.4 Role of m6A modification in endometrial receptivity

Endometrial tolerance refers to the ability of the endometrium to support embryo implantation. This tolerant state is the key factor of successful pregnancy. Increasing evidence has shown that m6A modification disorders are correlated with adverse pregnancy outcomes. This association is particularly evident in clinical diseases such as recurrent implantation failure (RIF) and abortion.

METTL3-mediated m6A modification is crucial to endometrial tolerance through a variety of regulatory mechanisms, and the homeostasis of METTL3 expression is also very important. Studies have shown that abnormal overexpression of METTL3 can reduce the stability of HOXA10 mRNA, leading to disordered expression of downstream tolerance-related genes (such as ITGB3 and EMX2), thus impairing the implantability of the embryo (115). Another study revealed that METTL3 is essential for maintaining normal endometrial tolerance through m6A-dependent progesterone receptor (PGR) translation regulation. The absence of METTL3 leads to a decrease in the level of PGR protein, causing hormone signal imbalance, abnormal cell proliferation and inflammatory reactions, ultimately damaging the tolerance of the endometrium (116).

Recent research has revealed cross-talk between m6A modification and histone modification in endometrial receptivity. Specific knockout of METTL3 reduces m6A modification on the transcript of EED, a core component of the key histone modification complex PRC2, thereby weakening the binding affinity of the reader protein YTHDC1 to EED mRNA. This leads to decreased levels of the repressive histone marker H3K27me3 and altered chromatin accessibility, ultimately causing dysregulation of critical receptivity genes (such as LIF, HOXA10, and HAND2) (117). This work elucidated for the first time the pivotal role of the METTL3-EED-YTHDC1 regulatory axis in modulating endometrial receptivity, providing a novel strategy for targeted RIF therapy and improving endometrial receptivity. In addition to METTL3, its partner protein METTL14 is also indispensable. METTL14 mediates m6A modification on SLC39A14 mRNA and reduces its expression, thereby facilitating the growth of endometrial stromal cells, inhibiting their apoptosis and autophagy, and ultimately improving endometrial receptivity (118).

Current research in this field focuses primarily on the core components of the m6A methyltransferase complex, such as METTL3 and METTL14, and preliminarily reveals their downstream target genes and signalling pathways. However, the roles of reader and eraser proteins in endometrial receptivity remain insufficiently explored and warrant broader investigation in the future.

## 6 Role of m6A in pregnancy maintenance and foetal development

As a core epitranscriptome mechanism, m6A modification regulates pregnancy maintenance and foetal development. At the maternal level, m6A acts as the foundation for the establishment and maintenance of pregnancy through the regulation of tumour function, vascular remodelling and reproductive endocrine homeostasis. At the foetal level, m6A guides the differentiation of embryonic stem cells and activates organ-specific development, thus ensuring the normal formation and development of key organs such as the nervous system, haematopoietic system, and heart. Therefore, precise regulation of the m6A pathway is crucial for a normal pregnancy and healthy foetal development, and m6A dysregulation is closely related to a variety of pregnancy complications and developmental defects.

### 6.1 m6A in trophoblast invasion and vascular remodelling

Trophoblasts serve as the core functional units of the foundation and continuation of pregnancy. Normal invasive capacity is a prerequisite for the remodelling of uterine spiral arteries and ensuring maternal–foetal material exchange. m6A modification plays a pivotal role in trophoblast invasion and vascular remodelling by dynamically regulating gene expression.

Dysregulation of m6A contributes to the pathology of PE by modulating trophoblast invasion and vascular remodelling. Evidence indicates that downregulation of WTAP expression reduces m6A modification on the mRNA of the chromatin regulator HMGN3, which impairs the binding of the reader protein IGF2BP1. Consequently, HMGN3 expression is decreased, disrupting the normal invasive program of trophoblasts and directly leading to shallow placental implantation and impaired vascular remodelling, thereby contributing to the onset of early-onset preeclampsia (ePE) (119).

More in-depth research has highlighted the critical importance of the m6A–YTHDC1–FGF2 regulatory axis. In normal trophoblasts, the METTL3/METTL14 complex catalyses m6A modification on FGF2 mRNA, which is recognized by the nuclear reader YTHDC1. YTHDC1 subsequently recruits the histone demethylase KDM3B to the FGF2 gene locus, activating FGF2 transcription by reducing the level of the repressive histone mark H3K9me2. The produced FGF2 protein, via autocrine/paracrine signalling, activates pathways such as MAPK/PI3K, thereby driving trophoblast migration, proliferation, and invasion, and ensuring successful spiral artery remodelling (120). Conversely, m6A deficiency leads to dysfunction of this pathway, impaired FGF2 expression, severely compromised trophoblast invasiveness, and ultimately the clinical symptoms of preeclampsia (120). This discovery provides the first *in vivo* evidence for a novel mechanism whereby m6A influences gene transcription and governs trophoblast function via histone modification.

Furthermore, m6A dysregulation is also implicated in abnormal trophoblast invasion in other pregnancy disorders, such as foetal growth restriction (FGR) and miscarriage. In FGR, the METTL3-IGF2BP2 axis stabilises FOSL1 mRNA through m6A modification. The subsequent upregulation of FOSL1 directly inhibits trophoblast invasion and migration, ultimately leading to placental insufficiency and FGR (121). In unexplained recurrent spontaneous abortion (URSA), METTL14 and IGF2BP1 drive trophoblast migration and invasion by regulating the expression of AQP3, which modulates the PI3K/AKT signalling pathway. Knockdown of either METTL14 or IGF2BP1 impedes this process, thereby contributing to the pathology of URSA (122).

Environmental pollutants and other external factors can also impair trophoblast function by disrupting m6A homeostasis. For instance, BPDE can activate the lnc-HZ01/MXD1 feedback loop, increasing METTL14-mediated m6A modification levels, which in turn suppresses trophoblast proliferation and promotes apoptosis—a mechanism identified as contributing to miscarriage (123). Similarly, prenatal arsenic (As) exposure reduces intracellular SAM levels, inhibiting m6A methyltransferase activity. This leads to decreased m6A modification on CYR61 mRNA and reduced binding by IGF2BP2, ultimately resulting in downregulated CYR61 expression, impaired EMT, and restricted trophoblast invasion, placental insufficiency, and FGR (124).

### 6.2 m6A in hormone secretion and metabolic regulation

The precise regulation of female reproductive hormones across the hypothalamic–pituitary–ovarian (HPO) axis is fundamental to reproductive cyclicity and fertility. Recent studies emphasise that m6A modification serves as a critical epitranscriptomic mechanism that fine-tunes this complex endocrine network (125, 126).

m6A orchestrates the release of gonadotropin-releasing hormone (GnRH) and gonadotropins. The demethylase FTO acts as a critical regulator in this process. It has been demonstrated that FTO-mediated m6A demethylation stabilizes Foxp2 mRNA, resulting in the upregulation of the cAMP/PKA signalling pathway and thereby promoting the synthesis and secretion of follicle-stimulating hormone (FSH) and LH (127). This regulatory layer is further supported by m6A-seq profiling of GnRH-stimulated pituitary, which identified thousands of dynamically modified transcripts, providing a molecular landscape for m6A-mediated control of hormone secretion (128). Moreover, FTO influences GnRH neuronal activity by regulating the processing of GABAAR mRNA in the hypothalamus. Inhibition of FTO reduces GABAAR protein expression, leading to aberrant GnRH secretion, impaired gonadotropin synthesis, and consequently, delayed puberty (129).

At the ovarian level, m6A is integral to steroid hormone signalling and the pathogenesis of endocrine disorders such as PCOS. FTO is recognised as a central regulator of hyperandrogenism in PCOS, where it modulates androgen metabolism and granulosa cell function by regulating the AR/AKT signalling pathway through m6A demethylation (130). Moreover, the methyltransferase METTL3 is crucial for maintaining the balance between oestrogen and progesterone signalling. Conditional knockout of METTL3 increases the stability of oestrogen target genes (e.g., Elf3 and Celsr2), resulting in hyperactivation of oestrogen signalling coupled with decreased progesterone receptor (PR) expression. This imbalance leads to progesterone resistance and, ultimately, infertility (131).

m6A modification is vital for establishing the endometrium for implantation. METTL3-mediated m6A modification regulates the translation of progesterone receptor (Pgr) mRNA, thereby fine-tuning progesterone signalling, which is critical for embryo implantation (116). In parallel, METTL14 facilitates embryo implantation through an m6A-dependent mechanism linked to ERα signalling (112).

In summary, m6A modification exerts multilevel control over the female reproductive endocrine system. Further investigation into its precise functions will expand our understanding of reproductive physiology and open new avenues for diagnostic and therapeutic strategies for endocrine-related reproductive diseases.

### 6.3 m6A in foetal organ-specific developmental programming

m6A modification lays the groundwork for subsequent organ-specific development by precisely regulating the lineage differentiation of embryonic stem cells (ESCs) into the three germ layers. A core mechanism involves promoting the degradation of mRNAs encoding core pluripotency factors, such as Nanog and Sox2, thereby driving cells to exit the self-renewal state and priming them for differentiation (132). Building on this foundation, m6A further directly mediates the initiation of organ-specific developmental programs by regulating key lineage-determining factors within each germ layer. During endodermal organ development, m6A precisely modulates Wnt signalling through the ALKBH5-GATA6 axis.

In hESCs with specific knockout of the ALKBH5 gene, the mRNA of the transcription factor GATA6 is recognized and degraded by YTHDF2 due to its aberrantly elevated m6A modification. The subsequent downregulation of GATA6 inhibits the expression of Wnt pathway antagonists DKK1 and DKK4, leading to overactivation of the Wnt/β-catenin signalling pathway and ultimately impairing the normal differentiation of the definitive endoderm. This mechanism ensures the correct initiation of the developmental program for organs derived from the endoderm, such as the thyroid, lung, liver, and pancreas (133).

Both the m6A reader protein YTHDF2 and the writer enzyme METTL14 play crucial roles in nervous system development. YTHDF2 ablation impairs neural stem cell self-renewal and disrupts neurogenesis in the embryonic neocortex, ultimately culminating in cell death due to the loss of precise spatiotemporal patterning (134). Further mechanistic studies demonstrated that this activity is related to the direct recognition and degradation of mRNAs encoding key neurodevelopmental genes, such as the axon guidance receptor ROBO1. The absence of YTHDF2 significantly downregulates the expression of ROBO1 and downstream neuronal differentiation genes (e.g., NEUROG1 and EOMES), thereby inhibiting neurodevelopment (135). Additionally, METTL14 destabilises the transcripts of the histone-modifying enzymes CBP and p300, resulting in a global increase in H3K27ac, which triggers abnormal proliferation and premature differentiation of neural precursor cells (NSPCs). This ultimately prevents the proper generation of neurons in the embryonic cerebral cortex (136).

Haematopoiesis is a precisely regulated process involving the coordinated action of multiple factors to ensure the correct commitment and maturation of blood cells. Accumulating evidence has demonstrated that m6A modification is pivotal to foetal haematopoiesis because it influences haematopoietic stem cell (HSC) fate decisions and cell maturation. YTHDF2, by regulating the stability of a wide range of mRNAs, is essential for preserving HSC homeostasis and stress haematopoiesis (137). A recent study revealed that knockout of METTL3 triggers the accumulation of nuclear dsRNA and an acute inflammatory response. While this promotes the expansion of megakaryocyte progenitors, it concurrently impairs the terminal maturation of megakaryocytes by suppressing the expression of insulin-like growth factor 1 (IGF1), causing reduced platelet production (138). These findings reveal a novel mechanism of m6A in balancing inflammatory stress and foetal haematopoiesis.

Furthermore, m6A is essential for normal cardiac morphogenesis and functional maintenance during the foetal period. Recent research suggests that the disparity between the transcriptome and the proteome in foetal versus adult hearts hints at a regulatory role for the epitranscriptome in cardiac biology. Conditional deletion of METTL3 or METTL14 in mice results in structural abnormalities in the foetal heart, which manifest after birth as severe ventricular dilation and early-onset dilated cardiomyopathy, underscoring the fundamental role of m6A in heart development (139).

## 7 m6A in the maternal-foetal immune microenvironment

The decidua at the maternal-foetal interface contains specialized immune cells, encompassing T cells, decidual natural killer (dNK) cells, macrophages, and dendritic cells (140). Following its progesterone-mediated formation from the endometrium, the decidua serves as a specialized mucosal niche that supports embryo implantation and enables placental development during early pregnancy (141). During the first trimester, immune cells account for 30%–40% of decidual cells. They perform critical roles by establishing maternal-foetal immune tolerance, facilitating placental development, and protecting against pathogenic threats (142).

**Figure 5:**
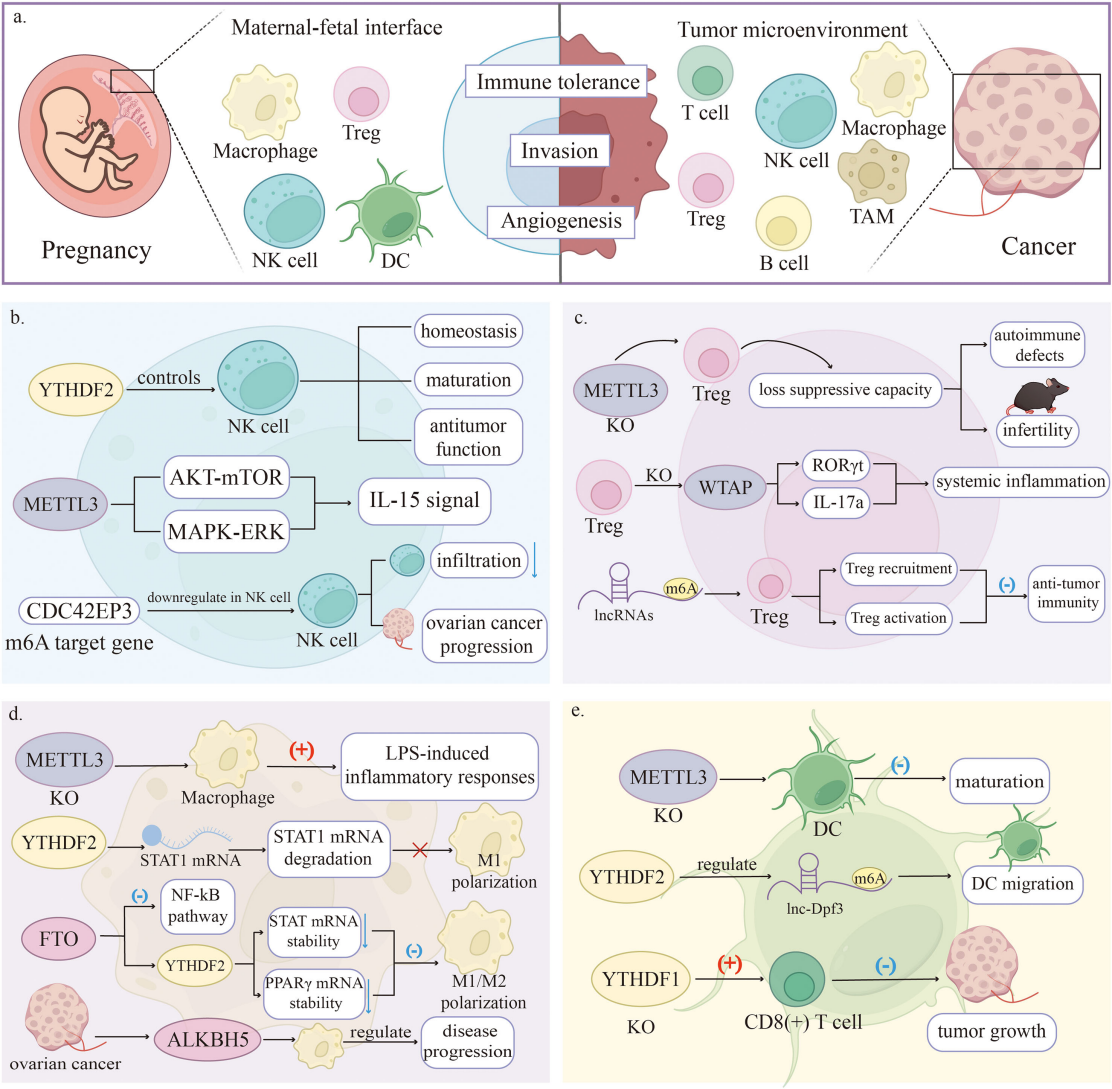
Potential regulatory roles of m6A methylation in the maternal-foetal immune microenvironment. **(A)** The maternal-foetal interface shares immunological similarities with the tumour microenvironment. **(B)** Tumour model studies indicate that m6A modulates NK cell homeostasis and function, suggesting that m6A may possess similar regulatory potential for dNK cells within the maternal-foetal immune microenvironment. **(C)** Specific knockout of the METTL3 or WTAP genes in Treg cells resulted in autoimmune deficiency and infertility in mice, indicating that normal m6A modification is essential for pregnancy. **(D)** m6A may regulate the balance between M1 and M2 phenotypes by modulating mRNA stability in key signaling pathways. However, this mechanism remains to be validated. **(E)** m6A is essential for DC maturation, migration, and inflammatory responses. Similar m6A-related mechanisms may guide DC establishment of maternal-foetal tolerance. (DC, dendritic cell; dNK, Decidual NK; TME, tumour microenvironment; Tregs, Regulatory T cells).

m6A modification has been shown to regulate immune cell differentiation and functional regulation, and plays a significant role in shaping the tumour immune microenvironment (143, 144). Research has confirmed that the maternal-foetal interface shares remarkable similarities with the tumour microenvironment in several key aspects, such as immune tolerance towards semi-allogeneic antigens, invasive growth properties, and the presence of an immunosuppressive state (145). However, the former represents a physiological process, while the latter constitutes a pathological process. Current understanding of the specific mechanisms of m6A in the maternal-foetal immune microenvironment remains incomplete. Nevertheless, based on its established functions and mechanisms in immune regulation and tumour immunology, we can reasonably infer that m6A may regulate the balance of the maternal-foetal immune microenvironment. (**Figure 5A**).

### 7.1 m6A in NK cells

Uterine natural killer (uNK) cells are a unique kind of immune cell residing in the endometrium and are responsible for maintaining immune homeostasis at the maternal-foetal interface. dNK cells, a specific uNK subset, represent the predominant immune cell population at the maternal-foetal interface in early pregnancy (146, 147). Unlike the potent cytotoxicity exhibited by peripheral blood NK cells, dNK cells play a key role in maintaining homeostasis at the maternal-foetal interface. They primarily function through the secretion of various growth factors and angiogenic mediators, including VEGF-C and IL-8. These factors facilitate critical processes such as spiral artery remodelling and trophoblast invasion (148, 149). The function of m6A modification in regulating NK cell bioactivity is a current research focus, and its interaction with NK cells in pregnancy-related diseases warrants further exploration.

Recent studies in tumour immunology have revealed that m6A modification regulates conventional NK cell function. For instance, YTHDF2 controls NK cell homeostasis, maturation, and antitumour function by modulating the mRNA stability of m6A-modified RNAs. Its absence inhibits the antitumour properties of NK cells (150). Furthermore, METTL3 deficiency impairs NK cell antitumour function by disrupting m6A-dependent homeostasis. Tumour-derived TGF-β suppresses METTL3, which attenuates IL-15 signalling via reduced m6A methylation in the AKT-mTOR and MAPK-ERK pathways, thereby reducing NK cell proliferation and granzyme B production (151). Bioinformatics analysis indicated that CDC42EP3, an m6A target gene, is downregulated in NK cells. This downregulation correlates with reduced NK cell infiltration and ovarian cancer progression (152) (**Figure 5B**).

These findings, while derived from cancer models, reveal universal mechanisms of m6A in NK cell immunoregulation, providing an analogical basis to elucidate the connection between m6A modification and dNK cells at the maternal-foetal interface. However, given the significant functional differences between dNK cells and conventional NK cells, it is not reasonable to directly extrapolate the mechanism based on the tumour model. Future studies are needed to investigate whether m6A modification regulates dNK-specific genes and functions in pregnancy-specific models.

### 7.2 m6A in T lymphocytes

At the maternal-foetal interface, decidual CD4+ and CD8+ T cells, particularly regulatory T (Tregs) cells, are crucial for a successful pregnancy by maintaining immune balance (153, 154). Tregs, an immunosuppressive T-cell subset, are critical for preventing foetal immune rejection. Their functional dysfunction is strongly linked to pregnancy complications, including recurrent spontaneous abortion (RSA) and infertility (155, 156).

Direct evidence from pregnancy-relevant models demonstrates that Treg-specific knockout of METTL3 causes a loss of their suppressive capacity, resulting in autoimmune defects and infertility in mice (157). This finding establishes a direct link between m6A modification and Treg-mediated maternal-foetal immune tolerance.

Beyond this direct evidence, studies in other immunological contexts provide insights into potential mechanisms that may operate at the maternal-foetal interface. In cancer models, m6A-associated lncRNAs have been shown to promote Treg recruitment or activation by modulating the immune microenvironment (158). In ovarian cancer, the C3a anaphylatoxin chemokine receptor (C3AR1) mediates T cell activation via m6A modification (159) (**Figure 5C**). Although these findings are derived from tumour models, they reveal universal mechanisms of m6A in T cell immunoregulation, providing a theoretical basis for hypothesizing potential roles at the maternal-foetal interface.

Furthermore, m6A regulates T cells function through broader mechanisms. Emerging evidence shows that conditional METTL3 knockout in mice prevents normal homeostatic expansion and differentiation of naive T cells (160). Moreover, m6A deficiency enhances the stability of SOCS mRNA and its protein expression, which suppresses the IL-7/STAT5 signal pathway and helps maintain naive T cells in a quiescent state (157, 160). This mechanism, which prevents excessive T cell activation, represents a potential pathway for averting maternal immune rejection of the foetus, although confirmation in decidual T cells is required.

### 7.3 m6A in macrophages

Decidual macrophages constitute the second most abundant immune population in early human decidua. These cells orchestrate immune homeostasis at the maternal-foetal interface through diverse functional pathways (161). Macrophages polarize into two main subsets: the pro-inflammatory M1 phenotype and the immunoregulatory M2 phenotype, each characterized by distinct functional programs (162, 163). Within the gestational microenvironment, an imbalance between M1-like and M2-like macrophages can disrupt maternal-foetal immune tolerance and contribute to the pathogenesis of pregnancy-related disorders (164, 165). Decidual macrophages function as crucial immunomodulators for sustaining normal pregnancy by directly suppressing maternal immune responses against the foetus by releasing anti-inflammatory cytokines such as IL-10 and CCL2 (166), by facilitating trophoblast invasion (167), and by facilitating foetal growth (168).

Studies from diverse pathological backgrounds, including inflammatory diseases and tumour models, have demonstrated the pivotal role of m6A modification in regulating macrophage polarization. In the context of inflammatory regulation, METTL3 depletion was found to exacerbate LPS-induced inflammatory responses in macrophages. This effect is achieved by impeding the YTHDF1/2-mediated degradation of NOD1 and RIPK2 mRNAs (169). Concurrently, YTHDF2 was demonstrated to restrain M1 polarization by facilitating the degradation of STAT1 mRNA (170). FTO ablation exerts a bidirectional inhibitory effect on both M1 and M2 polarization (171).

Although direct evidence validating m6A-mediated regulation of macrophages in pregnancy models remains scarce, studies from fields such as tumour immunology suggest context-specific regulatory roles. For instance, METTL3 has also been reported to enhance STAT1 mRNA stability by m6A modification, thereby orchestrating M1 macrophage polarization and suppressing M2-associated genes (172, 173). Similarly, METTL14 modulates macrophage function and polarization by regulating cancer cell glycolysis and lactate production (174). In ovarian cancer, tumour-associated macrophages influence disease progression via ALKBH5 expression levels (175) (**Figure 5D**).

These observations, derived primarily from non-pregnancy models, suggest that m6A modification is a central regulator of macrophage polarization, yet its specific role in decidual macrophages remains unexplored. Given the critical function of decidual macrophages in maintaining maternal-foetal immune tolerance, investigating how m6A modification shapes their identity and function in pregnancy-specific contexts represents a critical priority for future research.

### 7.4 m6A in DC cells

Dendritic cell (DC), a type of antigen-presenting cell (APC), orchestrates immunoregulation at the maternal-foetal interface (176, 177). In murine models, DC deficiency impairs implantation, reduces reproductive efficiency, causes decidual vascular malformation, and consequently affects placental development and pregnancy (178). This suggests that DC performs important immunomodulatory functions in the maternal-foetal microenvironment.

Studies in immunological and tumour microenvironments have revealed that m6A modification regulates DC function. Ket al. (179) showed that m6A modification decreases Toll-like receptors (TLRs) expression mediated by DC (e.g., TLR3, TLR7, and TLR8), suggesting that m6A modification affects innate immune responses. METTL3 knockout in DC impairs their maturation and compromises T cell stimulation (180). Regarding context-specific regulation at the maternal-foetal interface, YTHDF2 influences DC migration by modulating the stability of lnc-Dpf3. CCR7 stimulation reduces the m6A modification level on lnc-Dpf3, diminishing its YTHDF2-mediated degradation and leading to rapid lnc-Dpf3 accumulation, which subsequently suppresses DC migration and inflammatory responses (181). In the tumour microenvironment, YTHDF1 suppresses antitumour immunity by modulating lysosomal protease translation in DC (182) (**Figure 5E**).

These findings demonstrate that m6A modification serves as a master regulator of DC maturation, function, and immunoregulation in various pathological contexts. However, whether analogous mechanisms contribute to the establishment and maintenance of immune tolerance at the maternal-foetal interface remains to be directly investigated. Future studies should focus on elucidating the specific regulatory networks of m6A in pregnancy-associated DCs using gestational models.

### 7.5 m6A and immune in pregnancy process

The regulation of the immune system during pregnancy is a dynamic, precise, and spatiotemporally specific process. To ensure the survival and development of the semi-allogeneic foetus within the mother, the maternal immune system is neither simply suppressed nor activated but instead exhibits distinct functional states at different stages.

During embryo implantation, the maternal immune system displays a controlled pro-inflammatory state. This Th1-dominant cytokine environment is crucial for inducing trophoblast invasion, vascular remodelling, and the establishment of maternal-foetal immune tolerance, serving as an essential prerequisite for successful implantation and placentation (183). Entering the second trimester, to maintain pregnancy and protect the foetal from inflammatory damage, the maternal immune environment shifts towards an anti-inflammatory state. At this stage, a Th2-dominant immune milieu is established, and macrophages polarize towards an immunoregulatory M2 phenotype to enhance tolerance to the increasing load of foetal alloantigens (184, 185). Concurrently, the number of regulatory T cells significantly increases in the mother, systemically reinforcing immune tolerance to foetal antigens (186).

In late gestation, the immune system switches back to a pro-inflammatory mode to initiate parturition (187). For instance, in a horse pregnancy model, near term, the immune environment undergoes a significant shift from anti-inflammatory to pro-inflammatory, characterized by elevated IFN-γ and TNF-α levels, which activate immune pathways promoting placental separation and labour (188). Furthermore, during term labour, macrophages accumulate abundantly in the cervix, decidua, and myometrium. Responding to signals from placental aging and foetal lung maturation, they facilitate uterine contractions and cervical remodelling by secreting pro-inflammatory cytokines, matrix metalloproteinases, and prostaglandins (189).

m6A modification participates in shaping the immune microenvironment at the maternal-foetal interface by regulating the mRNA stability and translation efficiency of key cytokines and chemokines in immune cells and trophoblasts. Although no studies have directly addressed m6A-mediated regulation of the dynamic immune processes in pregnancy, m6A modification has been established as a critical regulator of immune cell functional states in other physiological and pathological systems. For example, METTL3 deficiency promotes the production of pro-inflammatory cytokines (e.g., TNF-α, IL-6) in macrophages (169), while FTO deficiency inhibits both M1 and M2 polarization (171). This suggests that m6A, through its various regulators, may precisely control the switch of immune cells between pro-inflammatory and anti-inflammatory states, thereby adapting to the tissue remodelling required during implantation and the immune tolerance needed for mid-gestation.

Furthermore, T cell-specific deletion of METTL3 leads to severe autoimmune disease in mice (157), further demonstrating that m6A is indispensable for maintaining the suppressive function of T cells, particularly Tregs. Given that Tregs are central to maintaining maternal-foetal immune tolerance, it is plausible to hypothesize that aberrant m6A modification in Tregs could lead to their functional impairment, rendering them unable to effectively protect the foetus from maternal immune attack, and thereby potentially contributing to pregnancy complications such as recurrent pregnancy loss.

Although these inferences are primarily drawn from research in non-gestational systems, they provide important clues regarding the potential role of m6A in the dynamic immune regulation of pregnancy. The specific impacts of m6A-mediated immune functions on the gestational process require further experimental validation and in-depth investigation.

## 8 m6A in pregnancy-related diseases

The homeostasis of the immune microenvironment at the maternal-foetal interface is critical for maintaining a normal pregnancy. Trophoblasts and endometrial stromal cells, among others, interact through complex molecular networks to collectively regulate embryo implantation, development, and maternal-foetal immune tolerance. In recent years, RNA methylation modification, particularly m6A modification, has been widely recognized to be involved in the regulation of cellular functions and immune homeostasis. In pregnancy-related pathologies, dysregulation of m6A modification can lead to cellular dysfunction and immune imbalance at the maternal-foetal interface, thereby contributing to the pathogenesis of gestational diseases such as PE and RSA.

### 8.1 m^6^A in preeclampsia

PE is a severe pregnancy-specific disorder, with its core pathological mechanisms lying in placental insufficiency and systemic vascular endothelial injury. Trophoblasts, as the primary constituent cells of the placenta, are not only responsible for embryo invasion and implantation but also actively participate in regulating the immune microenvironment at the maternal-foetal interface. Recent studies have indicated that m6A RNA methylation plays a critical role in maintaining trophoblast function and immune homeostasis, and its dysregulation is closely associated with the pathogenesis of PE.

In PE, aberrant expression of m6A regulatory factors can directly lead to trophoblast dysfunction and the release of pro-inflammatory factors, thereby disrupting the immune balance at the maternal-foetal interface. For instance, the loss of METTL3 in human trophoblast stem cells disrupts epigenetic homeostasis, activates pro-inflammatory gene programs, and induces molecular characteristics highly similar to those observed in patients with preeclampsia (190). METTL14, by regulating FOXP1, promotes the secretion of pro-inflammatory cytokines such as IL-6 and IL-8 in trophoblasts while impairing their invasion and tube formation capabilities (191). Beyond trophoblasts, m6A modification in decidual tissue is also involved in the pathogenesis of PE. Low expression of ALKBH5 inhibits decidualization and trophoblast invasion through the m6A-CORIN-HuR axis, suggesting it may serve as a potential therapeutic target for PE (192).

### 8.2 m6A in recurrent spontaneous abortion

Two or more consecutive pregnancy losses are defined as RSA, which is also closely associated with immune microenvironment imbalance at the maternal-foetal interface. Although direct evidence linking specific m6A modifications to RSA is still accumulating, existing studies have confirmed that immune microenvironment dysregulation plays a critical role in the pathogenesis of RSA (193, 194).

At the maternal-foetal interface, endometrial stromal cells not only provide structural support for the embryo but also shape the local immune microenvironment through interactions with immune cells (195). Studies have found that overexpression of ALKBH5 in stromal cells leads to a significant decrease in their ability to secrete vascular endothelial growth factor (VEGF). This reduction in VEGF impairs macrophage recruitment and hinders their polarization toward the anti-inflammatory M2 phenotype (195). This m6A-mediated immune remodelling may disrupt maternal-foetal immune tolerance, ultimately contributing to the occurrence of RSA.

Furthermore, research suggests that aberrant m6A regulators can reshape the immune microenvironment at the maternal-foetal interface by influencing the expression of downstream gene networks, leading to disturbances in immune cell composition, function, and immune balance. This may ultimately trigger immune rejection of the embryo, resulting in recurrent spontaneous abortion (196). However, research in this field is still in its early stages, and the specific molecular mechanisms require further elucidation and validation.

## 9 The diagnostic and therapeutic potential of m6A modification

As a pivotal epitranscriptomic mechanism, m6A RNA methylation is fundamental to sustaining pregnancy homeostasis, and its dysregulation has emerged as an emerging paradigm for elucidating the pathogenesis of various pregnancy-related diseases. Accumulating evidence demonstrates the diagnostic value and therapeutic potential of m6A modifications in gestational diabetes mellitus, preeclampsia, recurrent spontaneous abortion, and foetal growth restriction. Disease-specific alterations in global m6A levels and the expression profiles of its regulatory factors offer a promising avenue for developing novel non-invasive or minimally invasive biomarkers. Although therapeutic strategies directly targeting m6A in the context of pregnancy are still in their infancy, Small-molecule inhibitors from oncology research, precise targeted delivery technologies, and natural active compounds with m6A-modulating functions provide valuable insights for developing future intervention strategies.

### 9.1 Diagnostic landscape of m6A modification

Dynamic m6A RNA methylation equilibrium is crucial for maintaining normal pregnancy, whereas m6A RNA imbalance is implicated in the pathogenesis of various gestational diseases. Recent studies have revealed that global m6A levels and specific regulatory factors (e.g., METTL3, METTL14, FTO, ALKBH5, and YTHDF1/2/3) undergo disease-specific alterations in GDM, PE, RSA, and FGR. These findings suggest that m6A modifications are highly promising for the development of novel diagnostic and prognostic biomarkers, potentially enabling early and precise detection through analyses of placental tissue or peripheral blood.

Evidence from specific gestational pathologies underscores this diagnostic potential. In GDM, m6A modification presents a complex regulatory network. Wang et al. (197) reported a widespread reduction in m6A modification in GDM placentas, with these changes enriched in the 3’-UTR and CDS regions of mRNAs and showing specific reduction on key GDM-related genes like INSR and IRS1, suggesting that measuring gene-specific m6A levels could be a precise diagnostic tool. Conversely, a recent study indicated that METTL3 was significantly upregulated in GDM placentas and high glucose-treated trophoblasts, where it triggered trophoblast pyroptosis by stabilizing CEBPB expression (198). These seemingly contradictory findings underscore the intricate nature of m6A regulation in GDM and indicate that future diagnostic strategies may require an integrated assessment of global modification levels and the expression profiles of key regulators.

In PE, the expression profiles of m6A regulatory factors also demonstrate potential diagnostic value. Research indicates that METTL3 protein expression is significantly upregulated in PE model rat placentas, while the expression of the related copper transporter SLC31A1 is markedly decreased (199). Therefore, there are differences in the expression levels or ratios of METTL3 and SLC31A1 between patients with preeclampsia and healthy pregnant women. However, the accuracy and clinical applicability of these differences still require further experimental verification.

In RSA, Wang et al. (196) employed a random forest model to identify a diagnostic signature comprising FMR1, METTL14, LRPPRC, and RBMX. A diagnostic model built on these four genes demonstrated good accuracy and clinical utility and holds promise for the prompt and reliable diagnosis and risk prediction of RSA. Furthermore, the demethylase ALKBH5 is involved in spontaneous abortion, albeit with tissue-specific roles. Although its overall expression is increased in the trophoblasts of RSA patients, it is reduced in extravillous trophoblasts (EVTs), which are critical for embryo implantation (200). ALKBH5 maintains pregnancy success by demethylating and stabilising SMAD1/5 mRNA. Thus, quantifying ALKBH5 expression or global m6A levels in tissue or blood samples could aid in the early identification of high-risk pregnancies (200).

In FGR, disruption of the WTAP-m6A-IGF2BP3 regulatory axis is a key mechanism. The expression of WTAP, IGF2BP3, and its target gene ANLN is significantly reduced in FGR-induced placentas, impairing trophoblast proliferation and placental vascularisation (201). Key molecules within this pathway represent potential molecular biomarkers for distinguishing women with FGR from those with normal pregnancies.

Despite the promising diagnostic landscape, the translation of m6A-based biomarkers from laboratory findings to reliable clinical tools faces several challenges. Current studies primarily establish correlations, and the precise regulatory mechanisms of m6A in these diseases require further elucidation through functional experiments and rigorous case–control studies. Moreover, diagnostically valuable m6A alterations are predominantly identified in placental tissue, the acquisition of which is invasive and typically occurs after delivery or abortion, precluding early diagnosis. Therefore, exploring the detection of m6A-related indicators in more accessible samples, such as peripheral blood or other body fluids, is crucial, although such research remains scarce. While several diagnostic models have been developed, their clinical efficacy and utility must be validated in larger, independent, prospective cohorts.

Outside the field of reproductive medicine, m6A-based biomarkers have entered the clinical validation stage. For example, a research study constructed an m6A-related LncRNA prognostic model based on the TCGA database, which was confirmed by ROC analysis to have a strong predictive ability for the survival rate of head and neck squamous cell carcinoma (202). Additionally, the serum m6A-miRNA biomarkers completed the training cohort (AUC = 0.979), internal validation (AUC = 0.976), and external validation (AUC = 0.936) in 14,965 samples, demonstrating excellent diagnostic performance (203). These examples indicate that although m6A modification shows potential, its clinical translation in pregnancy-related diseases still requires equally rigorous validation.

### 9.2 Therapeutic targeting of m6A modification

Despite the absence of mature therapeutic strategies directly targeting m6A for gestational diseases, exciting advances in oncology and immunotherapy provide valuable paradigms and tools for future exploration. Small-molecule modulators of m6A regulators, such as the METTL3 inhibitors STM2457 (204) and STC-15 (205), as well as FTO inhibitors such as MO-I-500 and R-2HG (206), have demonstrated significant antitumour potential in acute myeloid leukaemia and solid tumours. Notably, a phase I clinical trial of STC-15 (NCT05584111) was completed in 2024, and has shown promising efficacy in advanced solid tumours and leukaemia (207, 208). An overall summary of m6A targeted therapy strategy is shown in **Table 2**.

**Table 2:** Overview of m6A-targeting therapeutic strategies.

Target	Disease/model	Agent/intervention	Effect	Development phase	Reference
METTL3	TNBC	STM2457	Combination therapy with STM2457 and anti-PD-1 agents improves survival rates	Preclinical	(1)
	osteosarcoma	STM2457	Overcome cisplatin resistance in osteosarcoma	Preclinical	(2)
	CRC	METTL3-single guide RNA/STM2457	Potentiates the effect of antiPD1 therapy	Preclinical	(3)
	NAFLD-HCC	METTL3 KD/VNP-si METTL3/STM2457	Inhibiting METTL3 in combination with PD-1 blockade enhances the efficacy of immunotherapy	Preclinical	(4)
	Advanced solid tumor	STC-15	STC-15 was well tolerated as monotherapy; combination with anti-PD-1 is under evaluation (NCT06975293)	Phase 1b/2	(5)
FTO	Melanoma	FTO KD	FTO inhibition can reduce resistance to anti-PD-1 therapy	Preclinical	(6)
	Acute myeloid leukemia	FB23-2	In xenograft mouse models, FB23-2 can significantly inhibit the progression of acute myeloid leukemia	Preclinical	(7)
	lung adenocarcinoma	FB23-2	FB23-2, an FTO inhibitor, synergistically suppressed the growth of resistant cells in combination with osimertinib	Preclinical	(8)
	HCC	CS2	Sensitised HCC to anti-PD-1 therapy	Preclinical	(9)
ALKBH5	NSCLC	ALKBH5 KD	Lung cancer cells with high ALKBH5 expression exhibit increased sensitivity to PD-L1 therapy	Preclinical	(10)
	CRC	VNP-siALKBH5	Enhances the efficacy of anti-PD1 therapy	Preclinical	(11)
YTHDF1	CRC	VNP-siYTHDF1	Enhance the efficacy of anti-PD-1 therapy in colorectal cancer	Preclinical	(12)
	NASH-HCC	LNP-si YTHDF1	Synergistically decreased tumor burden	Preclinical	(13)
YTHDF2	Liver tumor	YTHDF2 KO	Hepatic YTHDF2 is required for the synergistic antitumor effect of oxaliplatin and anti-PD-1 combination therapy	Preclinical	(14)
IGF2BP1	Melanoma/ Ovarian Cancer	BTYNB	BTYNB inhibited proliferation of IMP1-containing ovarian cancer and melanoma cells	Preclinical	(15)
IGF2BP2	OSCC	CWI1-2	Disrupts EGFR and PI3K/AKT signaling pathways, demonstrating potent anti-oral squamous cell carcinoma activity	Preclinical	(16)

CRC, Colorectal Cancer; HCC, hepatocellular carcinoma; TNBC, Triple Negative Breast Cancer; KD, knockdown; KO, knockout; NAFLD, nonalcoholic fatty liver disease; NASH, Non-alcoholic steatohepatitis; NSCLC, Non-Small Cell Lung Cancer; OSCC, oral squamous cell carcinoma.

These Small-molecule inhibitors represent a pioneering class of epigenetic drugs, and their pharmacological profiles and target engagement strategies provide a blueprint for developing analogous therapeutics against gestational diseases. However, given the dual role of m6A modification, their application remains challenging. Beyond its involvement in the pathogenesis of gestational diseases, m6A is indispensable for embryonic development, placental morphogenesis, and pregnancy maintenance. Therefore, any pharmacological intervention during pregnancy must carefully consider its potential long-term foetal toxicity.

Target specificity represents a major hurdle. The m6A writers and erasers are pleiotropic enzymes that govern numerous transcripts. Systemic modulation of these regulators during gestation may disrupt the essential epigenetic balance at the maternal-foetal interface, thereby leading to foetal developmental abnormalities. The current lack of comprehensive developmental toxicology data for m6A-targeted modulators constitutes a critical translational barrier to their application in gestational diseases.

Considering the potential foetal toxicity, the systemic use of m6A-targeted modulators is unlikely to be accepted clinically. Therefore, the innovation of targeted delivery systems is a necessary condition for the application of m6A targeted treatment strategies in the pregnancy. A seminal study developed a placenta-targeted nanocarrier, CRNP, by modifying lipid nanoparticles (LNPs) with a CGKRK peptide. This system successfully achieved efficient delivery of rosiglitazone to placental tissue, significantly alleviating oxidative stress and clinical symptoms in a PE mouse model (209). This strategy serves as a feasible technological template for m6A-targeted therapies, enabling precise drug delivery to diseased placental tissues while significantly minimizing foetal exposure risk. Future development of m6A modulators for gestational diseases must be integrated with such placenta-targeted platforms and accompanied by systematic toxicity assessment to ensure the safe delivery of m6A-targeted therapeutics.

Growing evidence suggests that natural active ingredients can modulate the m6A pathway. For instance, gambogic acid induces ferroptosis in colorectal cancer cells by promoting METTL3 ubiquitination and degradation (210). Hesperidin has been shown to regulate autophagy and m6A levels in contexts related to GDM (211), whereas baicalin may improve granulosa cell function via the mTOR signalling pathway (212). Although these studies were conducted in cancer or ovarian models, they revealed the intrinsic capacity of these compounds to modulate m6A-related pathways, suggesting their unexplored potential for managing gestational diseases and improving reproductive outcomes. However, natural ingredients often exhibit multitarget properties, and their efficacy, safety, and specific mechanisms of action in the context of pregnancy require further validation.

In summary, the application of m6A-targeted therapies in pregnancy-related diseases remains in its infancy. However, breakthroughs in other fields have provided valuable insights for this area of research. Future work should prioritise comprehensive target validation to identify disease-specific m6A sites, along with conducting developmental toxicology studies on existing m6A modulators and developing placenta-specific targeted delivery systems to facilitate the clinical translation of this promising field.

## 10 Conclusion and future perspective

m6A modification, the predominant internal chemical modification in eukaryotic mRNA, is dynamically and reversibly orchestrated by writer, eraser, and reader proteins. This sophisticated posttranscriptional modification mechanism precisely controls gene expression by regulating RNA stability, translation, and splicing. A growing body of evidence has demonstrated the critical role of m6A in both physiological and pathological processes throughout pregnancy, including oogenesis, embryo implantation, endometrial receptivity, and foetal development. This review systematically outlines m6A regulators, detection technologies, and their regulatory networks across the continuum of pregnancy, discusses their potential mechanisms within the maternal–foetal immune microenvironment, and evaluates their diagnostic and therapeutic potential in pregnancy-related disorders. The findings provide novel insights into pregnancy-associated physiological and pathological processes from an epitranscriptomic perspective.

Despite rapid progress in the field, research on the role of m6A modification in pregnancy processes, the maternal-foetal immune microenvironment, and related diseases remains in its infancy, with three major bottlenecks widely recognized: insufficient causal evidence, technical limitations in detection, and difficulties in clinical translation. Based on a critical analysis of existing literature, we recommend that future research should pursue synergistic development in both priority mechanisms and m6A sequencing technologies, while simultaneously advancing translational applications.

The vast majority of studies remain confined to correlation analyses between m6A regulator expression and phenotypic outcomes, lacking direct functional causal evidence. This represents an urgent issue requiring resolution. Future investigations should precisely dissect downstream pathways and establish the direct pathogenic role of m6A modification in pregnancy disorders such as PE and FGR. In addition, various epigenetic modifications operate collaboratively within intricate regulatory networks, rather than functioning independently. Therefore, future research should aim to delineate the synergistic or antagonistic interplay between m6A and other epigenetic marks, including m5C, m1A, and histone modifications, throughout gestation. For instance, whether dysregulation of m5C or m7G networks synergizes with m6A aberrations to produce additive effects, thereby contributing to implantation failure, developmental abnormalities, or related gestational diseases, remains an open question. Addressing this issue will not only validate the fundamental biological significance of these regulatory networks but also provide novel insights into the pathogenesis of pregnancy-related diseases.

Secondly, limitations persist in detection technologies. Current mainstream m6A sequencing methods are largely incapable of resolving modification profiles in distinct cell subpopulations within the placenta or embryo at single-cell resolution. The placenta, a complex organ composed of diverse trophoblast subtypes and immune cells, urgently necessitates the development of single-cell m6A sequencing technologies to enable the construction of cell-type-specific modification maps across different gestational stages. Furthermore, pregnancy-related samples (e.g., placenta, early embryos) are difficult to obtain, scarce in quantity, and highly heterogeneous. Widely used techniques such as MeRIP-seq demand relatively high amounts of high-quality RNA. This high requirement, however, restricts the generation of high-resolution modification maps, particularly during critical phases like early implantation and within the maternal–foetal interface microenvironment. Future breakthroughs are likely to rely on the application of low-input m6A sequencing (e.g., ULI-MeRIP-seq), chemical-based absolute quantification (e.g., GLORI 2.0/3.0), single-cell m6A sequencing (e.g., scm6A-seq) in pregnancy research, as well as more precise gene-editing tools and well-designed clinical studies.

Clinical translation also faces multiple challenges, most notably the lack of prospective validation in biomarker studies and the fact that intervention strategies remain at the proof-of-concept stage. Existing clinical sample studies are predominantly small-scale in design. To advance clinical translation, large-scale, multicentre, prospective cohort studies are essential. In addition, although the m6A-targeting Small-molecule inhibitor (STM2457) has accumulated preclinical safety data in oncology, its application in pregnancy-related diseases remains entirely unexplored. Future efforts should prioritize the establishment of rigorous placenta- and foetus-specific safety evaluation systems, and actively leverage strategies such as drug repurposing and precision delivery to overcome these obstacles, ultimately enabling early clinical diagnosis and personalized therapy for pregnancy-related disorders.

In the future, breakthroughs in m6A research and its clinical translation are anticipated to drive a dual transformation in current diagnostic and therapeutic strategies. Traditional biomarkers typically become significantly elevated only during the clinical phase of disease, and placental tissue is obtainable solely after delivery, thereby precluding early prenatal warning. Recent studies have indicated that m6A modifications undergo dynamic changes during the early stages of pathological stress and can be detected in cell-free RNA derived from maternal peripheral blood. Consequently, assessing the global m6A modification abundance or gene-specific modification sites in plasma cell-free RNA holds promise for establishing risk prediction models for gestational diseases in the first trimester, offering a novel avenue for achieving genuine early warning. Nevertheless, this technology remains in its nascent research phase, and its clinical translation is confronted with challenges pertaining to technical standardization, detection sensitivity, specificity, and cost.

At the therapeutic level, the translational application of m6A research is poised to shift intervention strategies for pregnancy-related disorders from non-specific symptomatic management toward targeted epitranscriptomic regulation. Future efforts may leverage the experience garnered from the development of highly selective m6A modulators in oncology to screen candidate compounds suitable for pregnancy complications, thereby inaugurating new directions for precision intervention. For instance, in the context of preeclampsia characterized by inadequate trophoblast invasion and impaired spiral artery remodelling, the feasibility of localized placental delivery of METTL3 inhibitors could be explored to restore critical cellular functions during early gestation. However, this approach must still surmount key obstacles, including placental targeting efficiency, foetal safety assessment, and the pharmacokinetic specificities inherent to pregnancy. Moreover, with an increasingly comprehensive understanding of the regulatory roles of m6A modifications in pivotal pathways such as trophoblast invasion, spiral artery remodelling, and maternal–foetal immune tolerance, it is envisioned that the reprogramming of pathogenic gene expression programs may be achieved in the future, transcending mere symptomatic alleviation.

In summary, research centred on m6A modification has significantly advanced our understanding of the regulatory networks governing pregnancy. Overcoming current challenges through interdisciplinary collaboration holds promise not only for advancing m6A-based diagnostic models but also for establishing m6A-targeted intervention strategies as important future approaches for managing pregnancy health.
